# Systems biology of Alzheimer’s Disease: a scoping review of key pathways and mechanisms

**DOI:** 10.1186/s13024-026-00934-4

**Published:** 2026-02-23

**Authors:** Paula J. Bice, Kwangsik Nho, Nilüfer Ertekin-Taner, Andrew J. Saykin

**Affiliations:** 1 Center for Neuroimaging, Department of Radiology and Imaging Sciences, Indiana University School of Medicine, 355 W 16th St., Indianapolis, IN 46202 USA; 2 Indiana Alzheimer’s Disease Research Center, Indiana University School of Medicine, 355 W 16th Street, Goodman Hall Ste 4100, Indianapolis, IN 46202 USA; 3 Stark Neurosciences Research Institute, Indiana University School of Medicine, 355 W 16th St., Indianapolis, IN 46202 USA; 4 School of Informatics and Computing, Indiana University, 535 W Michigan St., Indianapolis, IN 46202 USA; 5 Department of Neurology, Mayo Clinic, Jacksonville, FL USA; 6 Department of Neuroscience, Mayo Clinic, Jacksonville, FL USA; 7 Department of Neurology, Indiana University School of Medicine, 355 W 16th St., Indianapolis, IN 46202 USA; 8 Department of Psychiatry, Indiana University School of Medicine, 355 W 16th St., Indianapolis, IN 46202 USA; 9 Department of Medical and Molecular Genetics, Indiana University School of Medicine, 410 W. 10th St., HITS 3000, Indianapolis, IN 46202 USA

**Keywords:** Alzheimer’s Disease, Biological pathways, Biomarkers, Systems biology, Multi-omics

## Abstract

Alzheimer’s Disease (AD) is a progressive and ultimately fatal neurodegenerative disorder, representing the most common form of dementia and affecting nearly 7 million people in the United States. For over a century, research has centered on hallmark AD pathologies—extracellular β-amyloid (Aβ) plaques and intracellular neurofibrillary tangles composed of hyperphosphorylated tau (pTau). While these mechanisms remain central to our understanding of the Disease, the underlying molecular events and broader biological pathways driving AD are still not fully understood. Precision medicine for AD and related dementias (ADRD) requires a comprehensive understanding of neurodegenerative Disease through pathophysiologic biomarkers for early detection, prediction of progression, and for evaluating response to treatment. Systems biology provides a powerful framework for this effort, leveraging multi-omics for biomarker discovery and elucidation of critical Disease pathways. The purpose of this scoping review is to identify and inform the field on key pathways being intensively investigated in AD/ADRD, including hallmark pathology, proteostasis, inflammation, oxidative stress, glucose metabolism, lipid dysregulation, neurotransmitter systems, synaptic integrity, neurogenesis, co-pathology- and sex-related pathways. Finally emerging biomarkers, tools and strategies for discovery and analysis are discussed, emphasizing advances related to the emerging precision medicine for AD.

**Graphical Abstract:**

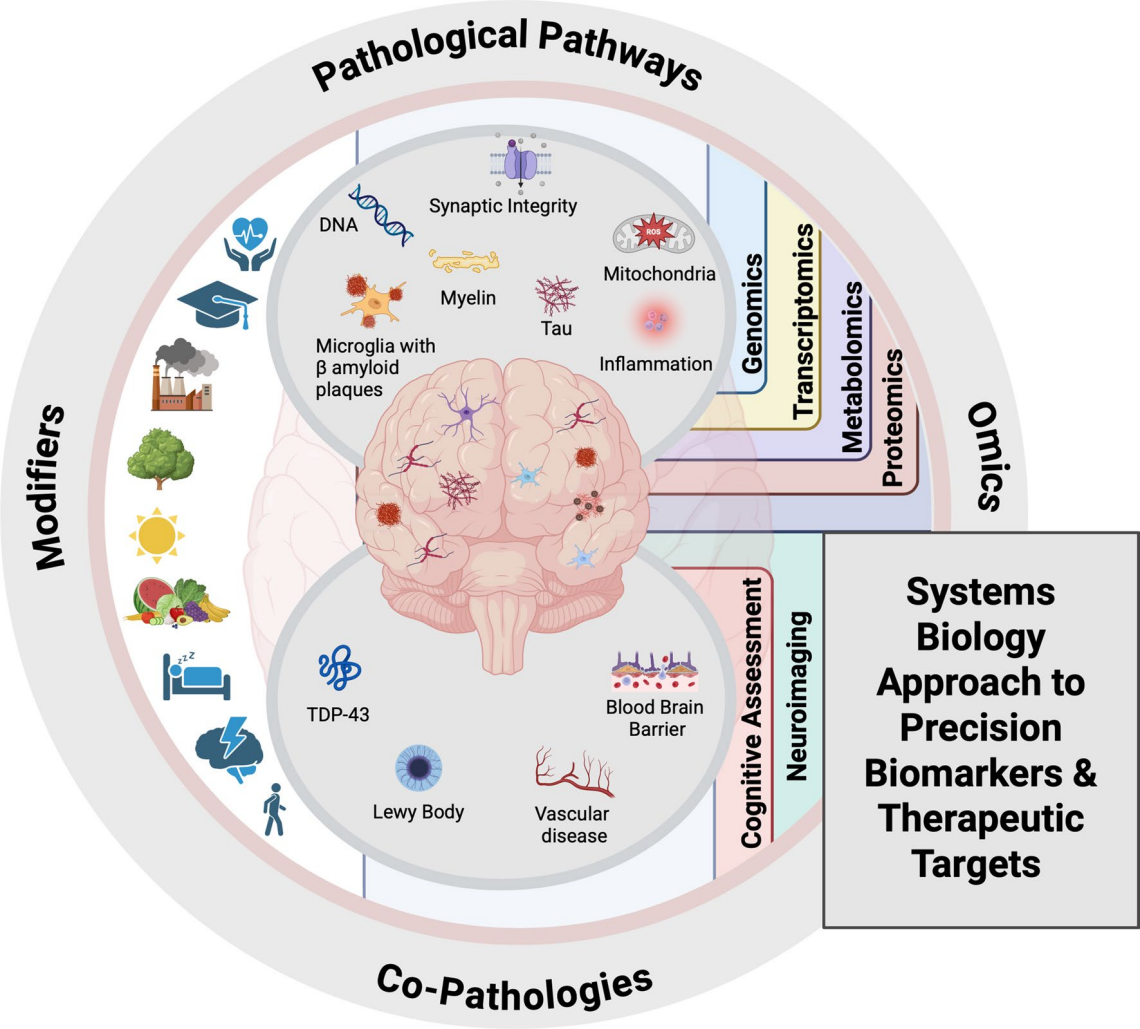

**Supplementary Information:**

The online version contains supplementary material available at 10.1186/s13024-026-00934-4.

## Background

### Alzheimer’s Disease

Alzheimer’s Disease (AD) is a progressive, neurodegenerative illness and the leading cause of dementia. It produces a severe decline in memory, executive function, language, behavior, and personality, leaving the patients in late Disease stages with the inability to perform even the most basic activities associated with daily living. Collaterally, it devastates family members and caregivers, physically, emotionally, and financially. An estimated 7.2 million people (age 65 and older) are affected by AD in the U.S., with the prevalence growing continually as the population ages [1]. Current statistics show that AD is the seventh leading cause of death in the U.S. and the sixth leading cause of death in those 65 or older [1]. While deaths from other Diseases, such as stroke and heart Disease have decreased in recent years, the prevalence of AD has increased by more than 142%, claiming more lives than breast cancer and prostate cancer combined [1].

### Hallmark pathophysiology

Research into the Disease processes of AD has focused primarily on hallmark pathologies, including extracellular β-amyloid (Aβ) plaque deposition (comprised mostly of Aβ_1–40_ and Aβ_1−42_ peptides), intracellular neurofibrillary tangles of hyperphosphorylated tau (pTau), and neurodegeneration [2], representing the **A/T/N** classification schema for research diagnosis and clinical studies [3, 4]. According to the amyloid cascade hypothesis, the pathophysiological events culminating in neurodegeneration in AD are proposed to be triggered by abnormal accumulation of Aβ plaques in various brain regions [5]. Aβ results from the cleavage of amyloid precursor protein (APP) by β- and γ secretases [6, 7]. Mutations in *PSEN1* or *PSEN2*, which code for presenilin 1 and 2, respectively, the major subunits of the γ secretase complex, determine the length of Aβ, with the longer forms, particularly hydrophobic Aβ_42_ [8], aggregating more readily [9], causing downstream effects that lead to the hyperphosphorylation of tau protein, which becomes paired helical filaments that ultimately form neurofibrillary tangles in the cell bodies of neurons [10–15], the apparent impetus for neuronal dysfunction and associated neurodegeneration that accompanies the development and progression of AD [16].

While Aβ and tau pathologies continue to be of paramount importance as therapeutic targets and biomarkers, they cannot fully explain the heterogeneity or polygenic nature of AD [17, 18]. In fact, two thirds of people in their 80’s who have plaques, do not exhibit clinical AD [19], and therapies that target Aβ aggregation and accumulation, although modestly slowing progression, have been ineffective in reversing the course of the Disease, primarily because Aβ accumulation occurs years or even decades prior to cognitive decline, by which time an individual may have sustained significant neurodegenerative changes [20]. Thus, exploring additional biological pathways that are candidate mechanisms for driving Disease progression is essential for advancing therapies and biomarker development for early detection of AD.

Given the extensive heterogeneity in AD, effective biomarker and therapeutic discovery will require a *systems biology* approach, an integrative strategy that combines data from multiple biological scales and layers to uncover complex molecular interactions. This includes the application of rapidly evolving multi-omics analyses, such as genomics, proteomics, transcriptomics, epigenomics, metabolomics, and microbiomics [21, 22], as shown in both human and AD models, as well as lifestyle and environmental factors that identify key molecular pathways involved in AD. This holistic approach, which leverages global molecular and exposomal profiling, aims to capture all elements affected by the Disease state – from the level of a single cell to entire brain regions and the whole organism – providing a broad and comprehensive understanding of Disease-relevant networks across a multimodal dataset that spans asymptomatic, subjective cognitive decline (SCD), mild cognitive impairment (MCI), and AD populations [23].

This review explores key biological pathways underlying AD/ADRD and highlights the potential of a systems biology approach in identifying genomic contributors and uncovering novel Disease-associated processes, thereby advancing biomarker discovery and paving the way for precision medicine strategies tailored to individual molecular profiles.

In the context of this review, a biological pathway refers to a defined series of molecular and cellular events that contribute to the development, progression, or modulation of AD/ADRD. These pathways encompass diverse, often overlapping mechanisms operating at multiple biological scales, from genes and proteins to cells, circuits, and systems, used to organize current knowledge about Disease biology. The framework for this review is informed by the CADRO (Common Alzheimer’s and Related Dementias Research Ontology) Category A classifications [24], which include, but are not limited to, key processes such as amyloid beta and tau pathology, presenilin biology, apolipoprotein E (APOE) and lipid neurobiology, autophagy and proteostasis, synaptic and circuit dysfunction, cell death, immune and inflammatory responses, metabolic and mitochondrial function, vascular and neuroendocrine mechanisms, neuroprotection, microbiome-host interactions, environmental exposures, and genetic and multi-omics contributions. Each pathway reflects a biological theme that can interact with others, forming an integrated network of mechanisms underlying AD pathogenesis and progression. The maturity of evidence across these domains varies considerably—while hallmark pathologies such as amyloid, tau, and neuroinflammation are supported by decades of convergent data, emerging systems-level areas such as microbiomics, exposomics, and integrative omics remain in earlier stages of validation. This review highlights both well-established and developing frameworks to contextualize the evolving landscape of AD biology and biomarker and therapeutic target discovery.

### Methodology: search strategy and study identification

This scoping review was conducted following the methodological framework outlined by Munn et al. [25] and reported in accordance with PRISMA-ScR guidelines [26]. The objective was to map the breadth of evidence across molecular, cellular, and systems-level biological pathways implicated in AD/ADRD, with attention to emerging biomarkers and therapeutic targets.

#### Search and data sources

A structured search of PubMed was performed to identify studies published in English between January 1, 2009 and December 31, 2024, a period of accelerated funding for AD research in the U.S. and globally. Searches were filtered by article type to include reviews, systematic reviews, meta-analyses, clinical trials, randomized controlled trials, observational studies, and preprints (gray literature).

Additional sources included four publicly available data or policy resources identified via Google (e.g., the NIA Alzheimer’s Disease Sequencing Project website) and one peer-reviewed study identified through Google Scholar. Twelve seminal studies published prior to 2009 were retained due to their foundational relevance to current AD biological frameworks.

Forty-eight search terms representing major CADRO biological domains were used, including amyloid-β, tau, proteostasis, autophagy, microglia, astrocytes, blood–brain barrier, oxidative stress, mitochondrial dysfunction, insulin signaling, lipid dysregulation, neurogenesis, neurotransmitter systems, synaptic integrity, vascular Disease, α-synuclein, TDP-43, sex differences and hormones, and multi-omics domains (genomics, epigenomics, transcriptomics, proteomics, metabolomics, microbiomics). Each term was combined with “Alzheimer*” using Boolean operators.

#### Inclusion and exclusion criteria

Studies were included if they: (a) addressed AD or ADRD as the primary condition; (b) examined at least one biological pathway corresponding to CADRO Category A classifications; and (c) reported molecular, cellular, or circuit-level or systems biology findings relevant to human AD pathology or biomarker/therapeutic development.

Exclusion criteria were: (a) studies limited to non-neurodegenerative conditions; (b) editorials, commentaries, or case reports lacking primary data; and (c) studies without mechanistic, biomarker, or pathway-specific relevance.

#### Record management and manual screening

**Fig. 1:**
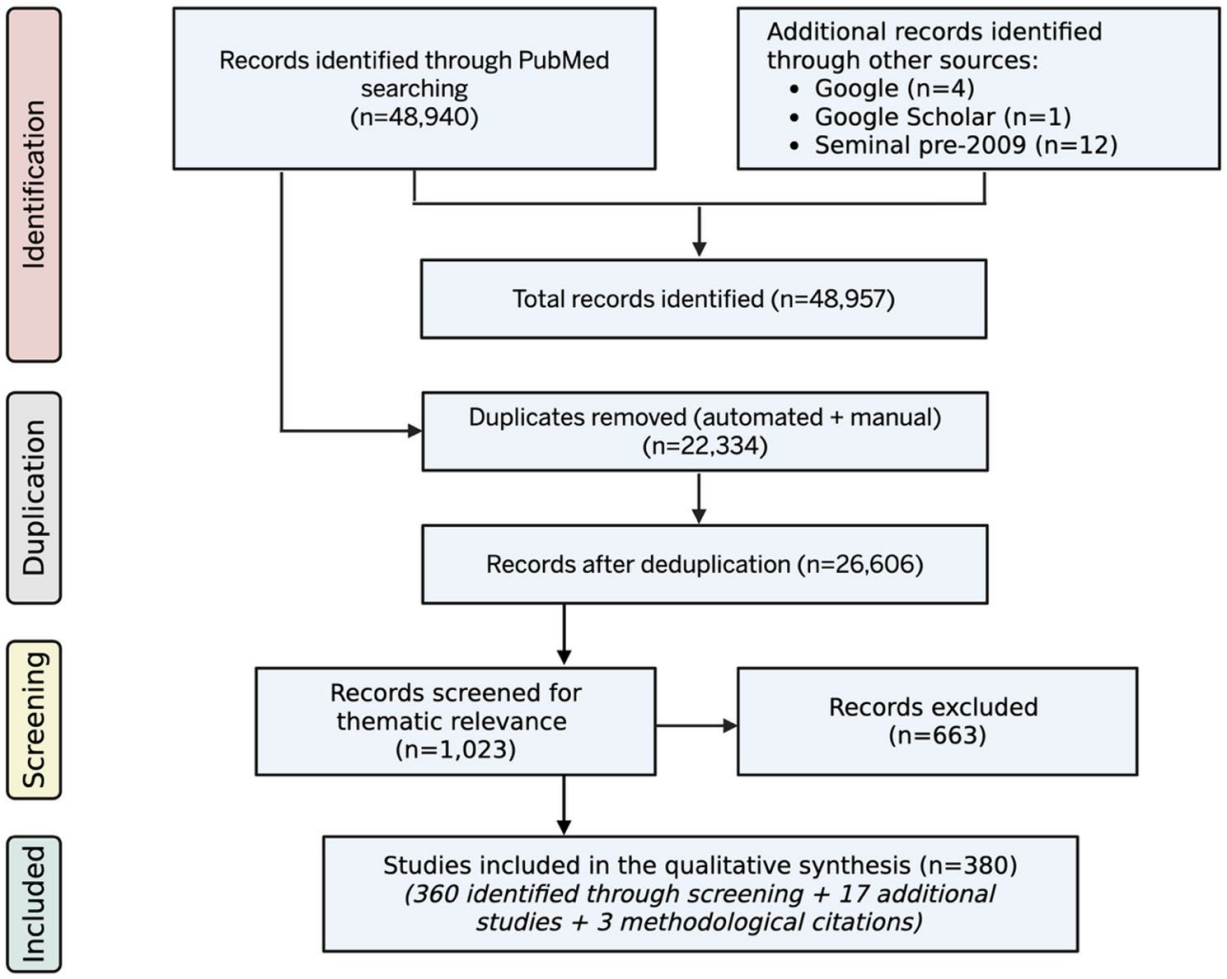
PRISMA-ScR flow diagram summarizing record identification, deduplication, thematic screening, and study inclusion. The diagram outlines the number of records identified through PubMed and additional sources, duplicates removed, records screened for thematic relevance, and studies included in the qualitative synthesis. Created in https://BioRender.com

The initial search retrieved 48,940 records. Following automated and manual de-duplication in EndNote (v 2025.1), 26,606 unique records remained. The records were thematically filtered and screened by title and abstract within a scoping-review framework to identify studies relevant to major biological domains. A summary of study identification, deduplication, and manual screening are shown in Fig. 1.

Full-text articles meeting inclusion criteria were examined for data extraction, capturing study design, biological pathway(s), molecular/cellular mechanisms, biomarker relevance, and therapeutic implications. A descriptive summary of study characteristics across the included literature is provided in Supplementary Table S1.

#### Integration of machine-learning–assisted screening (ASReview)

To evaluate the completeness of the manually curated corpus and enhance reproducibility, machine-learning–assisted screening was performed using ASReview LAB (v2.1.1) [27]. Five domain-specific datasets were created to reflect key biological themes in AD systems biology: Hallmark AD Pathology (amyloid-β, tau, plaques, tangles, staging, seeding); Copathology (TDP-43, α-synuclein, vascular, mixed dementia); Immune and Oxidative Stress Pathways; Synaptic Integrity and Neurotransmission; and Multi-Omics (genomics, transcriptomics, proteomics, metabolomics, microbiome).

Each domain dataset was screened using an active-learning model combining uncertainty sampling and relevance-based ranking. Screening proceeded until the recall curve stabilized, indicating minimal additional yield of newly identified relevant studies. Detailed ASReview metrics for each domain, including initial dataset size, screening depth, and relevant study counts are provided in Supplementary Table S2.

#### Concordance with manually identified references

To assess alignment between manual and machine-learning approaches, ASReview-identified relevant studies were compared with the references included in the manuscript. Overlap was observed across all domains, demonstrating strong concordance between the manual search strategy and machine-learning–assisted prioritization. These analyses demonstrate that ASReview functioned as a validation tool and helped identify additional literature in large, heterogeneous domains. Domain-specific overlap values are summarized in Supplementary Table S2.

### Proteostasis

While the pathological accumulation of misfolded proteins, particularly Aβ and pTau, is a defining feature of AD, less emphasis has been placed on the cellular systems responsible for maintaining protein homeostasis.

Maintaining proteostasis, the delicate balance between protein synthesis, folding, trafficking, and degradation, is critical for neuronal health [28]. Disruptions in this tightly regulated system contribute to the accumulation of misfolded proteins, a hallmark of AD [28, 29]. Among the key players in proteostasis are heat shock proteins (HSPs), molecular chaperones, which prevent protein misfolding, facilitate refolding, and promote the clearance of aggregated proteins [28]. Dysregulation of HSP activity in AD is implicated in both Aβ and tau pathologies, underscoring their relevance in neurodegenerative processes [28, 30].

#### HSPs in Aβ aggregation and clearance

HSPs, particularly HSP70 and HSP90, interact with Aβ peptides to prevent the formation of toxic oligomers and fibrils [28, 30–34]. HSP70 has been shown to suppress Aβ aggregation by binding to early oligomeric intermediates, guiding them toward degradation pathways such as the ubiquitin-proteasome system (UPS) and autophagy-lysosome pathways [28, 32, 35]. Similarly, HSP90 plays a dual role — while it assists in protein refolding, it may also stabilize misfolded proteins, inadvertently promoting the persistence of pathological aggregates [36].

#### HSPs and tau stabilization

HSPs also modulate tau homeostasis by promoting proper folding and preventing excessive phosphorylation. HSP70, in particular, facilitates tau degradation through chaperone-assisted selective autophagy [37–39], while HSP90 inhibitors have been shown to enhance tau clearance via the UPS [40, 41]. Dysregulation of these chaperone-mediated processes may contribute to tau accumulation and synaptic dysfunction in AD [28, 30].

#### HSPs and neuroinflammation

Neuroinflammation, characterized by microglial activation and cytokine release, is strongly associated with AD progression. HSPs, particularly HSP70, exhibit immunomodulatory effects by inhibiting NF-κB signaling, thereby reducing the production of pro-inflammatory cytokines [42–44]. This anti-inflammatory role of HSPs suggests they may act as protective modulators that help limit neuroinflammation-associated synaptic damage [43, 44].

#### HSPs as therapeutic targets in AD

Given their protective role in mitigating Aβ and tau pathology while also modulating neuroinflammation, HSPs have emerged as potential therapeutic targets in AD [30, 43]. Small-molecule HSP90 inhibitors, for example, are under investigation in preclinical and early-stage studies for their capacity to promote tau degradation and reduce neurotoxicity [30, 45]. However, fine-tuning HSP modulation remains a major challenge, as excessive inhibition can disrupt essential protein-folding processes and impair cellular proteostasis [30, 45].

Although dysregulation of HSPs contribute to both Aβ and tau accumulation [28], as well as to inflammatory signaling, efforts to translate HSP-based modulation into safe and effective therapies are still in an experimental phase. Future work aimed at selectively enhancing protective HSP functions while minimizing adverse effects may help determine whether this pathway can be harnessed for Disease-modifying benefit in AD.

Together, these findings underscore the central role of proteostasis in AD pathogenesis but also reveal uncertainty about which chaperone systems are primary drivers versus downstream responders. Differences across model systems and experimental conditions highlight the need for standardized approaches and longitudinal human studies to determine whether proteostatic failure precedes or follows protein aggregation.

### The immune system and inflammation in AD

The immune system plays a pivotal role in AD pathophysiology, with neuroinflammation emerging as a key mechanism driving Disease progression. The immune system, both *innate* and *acquired*, make up a complex network that protects the body from foreign invaders. Under acute conditions, inflammation is a necessary and beneficial response of the immune system, allowing it to pinpoint injury or infection and promote healing. These events are controlled by a host of immune regulators, including members of the cytokine and chemokine families, as well as growth factors, that recruit immune cells and control the immune response through complex signaling pathways [46, 47]. Under chronic conditions, inflammatory regulators have been shown to impact AD, particularly amyloid processing, Aβ aggregation, and tau phosphorylation [47, 48]. Inflammation (I) has also been proposed as a formal biomarker category for AD staging and diagnosis [4]. The regulators implicated in AD include pro-inflammatory cytokines interleukin-1 (IL-1β/α), IL-6, IL-18, tumor necrosis factor (TNFα), and Type 1 interferon (IFN); pro-inflammatory chemokine, IL-8, and anti-inflammatory cytokines IL-4, IL-10, and IL-12, where IL-12 also has pro-inflammatory activity [46–50]. These regulators, which are measurable in cerebrospinal fluid (CSF) or plasma, may represent early biomarkers of neurodegeneration. Modulation of inflammation and its regulators represents an area of growing interest for therapeutic exploration [47, 48]. However, the specific molecular targets, optimal timing within the Disease course, and direction of immune modulation that would provide clinical benefit remain unclear. Current approaches remain largely experimental, and further mechanistic and translational studies will be needed before inflammation-targeting strategies can be realized in human AD therapy.

#### Role of microglia

Activation of microglia, the brain’s resident immune cells, can have both protective and deleterious effects. Microglia help maintain brain homeostasis. In a normal response, microglia are recruited by the immune system to function as macrophages, where they clear debris and pathogens via phagocytosis [51]. However, under pathological conditions, microglia become overactive and release many types of cytotoxic molecules, including proinflammatory cytokines (IL-1β, IL-6, IL-18, TNFα and IFN, etc.), nitric oxide, superoxide, proteinases, matrix metalloproteinases (MMPs), and others, which exacerbate Aβ burden and perpetuate microgliosis [15, 47, 50, 52–54]. Sustained aberrant activation of microglia has been found to be a key factor in the progression and development of multiple neurodegenerative Diseases, including AD [55].

Indeed, both rare and common variants in microglial genes [56–59] have been identified as risk or protective variants for AD/ADRD. In general, variants that enhance microglial function [56, 60, 61] appear protective, whereas variants associated with reduced microglial activity or impaired phagocytic response increase [58, 59, 62–69]. Genetic studies have identified several microglial receptors and signaling pathways that influence AD susceptibility. Among the most established is triggering receptor expressed on myeloid cells 2 (TREM2), which regulates microglial activation, survival, and phagocytosis [70]. Rare missense variants in *TREM2* increase risk for late-onset AD by 2–3-fold [71–73], whereas regulatory variants that enhance its expression appear protective, underscoring its role in modulating neuroinflammatory responses [72]. Downstream of TREM2, the adaptor DAP12 and associated kinases mediate phagocytic and inflammatory signaling [72, 74]. In parallel, variants in phospholipase C-γ2 (PLCG2) further influence microglial phenotype and function, with *PLCG2*^*M28L*^ linked to increased risk plaque burden and *PLCG2*^*P522R*^ associated with attenuated pathology in preclinical models [75]. Together, these pathways highlight how microglial signaling diversity contributes to both risk and resilience in AD. Such findings underscore how genetic studies of immune regulation can clarify Disease mechanisms and identify potential molecular targets, although translating these insights into effective therapies will require substantial validation across experimental systems and clinical cohorts.

#### Role of astrocytes

Astrocytes are the largest and most abundant neuroglia in the CNS. There are various subtypes that, in combination, contribute to the microarchitecture of the brain parenchyma. Astrocytes are important for the integrity of the blood brain barrier (BBB) and provide mechanical and trophic support for neurons by controlling concentrations of ions, neurotransmission, and other metabolites. Astrocytes are also involved in neuronal cell development, neuroplasticity, synaptogenesis [76], and while they do not have an electrically excitable membrane, they are involved in complex Ca^2+^ signaling across astrocytic networks, thereby impacting the local effects of neuromodulation and potentially participating in real-time coding of information in the brain [77, 78]. A study by Shah et al. found that Ca^2+^ signaling, which has been shown to decrease several months prior to amyloid plaques development, disrupts neuronal regulation and may mitigate the initial features of early AD pathology [79]. Additionally, molecular studies in AD models have shown that astrocytes, like microglia, are involved in the innate immune response, where they regulate inflammatory factors, such as cytokines, chemokines, and reactive oxygen species (ROS), which contribute to neuronal death or other neural disturbances [80–83]. Studies in humans have also shown that late onset AD (LOAD) is affected by genes (e.g., *APOE*, *APOJ*, and *SORL*) that are expressed in glial cells, including astrocytes [81]. In addition, astrocytes react to CNS injury through astrogliosis and glial scar formation, exacerbating inflammation [84]. Whether astrocytes contribute to neurotoxicity through inflammatory signaling or are themselves impaired by neurodegeneration, disruption of their neuroprotective functions—such as maintaining the blood-brain barrier—can exacerbate neuronal damage and accelerate Disease progression. The role of astrocytic dysfunction in AD, including the presence of Disease-associated astrocytes in brain tissue, is being investigated through multi-omics studies reviewed elsewhere [85].

#### Role of the blood brain barrier

The BBB, as part of the neuroimmune axis [86], is a neurovascular unit formed by endothelial cells, astrocytes, pericytes, and the extracellular matrix. The function of the BBB is to maintain a homeostatic microenvironment and provide a dynamic interface between the brain and peripheral blood circulation [87, 88], thereby preventing microorganisms, toxins, etc. from invading the brain [89]. Molecules necessary for brain survival, such as nutrients, ions, organic anions, and macromolecules, enter either passively or via active transport [87]. BBB dysfunction is thought to be involved in the progression of AD, particularly through Aβ trafficking [90]. Aβ exchange is influenced by several BBB transporters that aid in clearance from the brain to peripheral blood circulation, including a subfamily of ABC transporters, of which ABCB1 (P-glycoprotein) has been shown to play a significant role in Aβ clearance [91]. Other transporters, including increased levels of RAGE (receptor for advanced glycation end products) and decreased levels of LRP1 (low density lipoprotein receptor-related protein-1) also cause dysfunction of Aβ transport in the BBB [90]. Further, under pathological conditions, the BBB becomes more permeable, increasing CNS vulnerability to Disease and pathogens [90, 92, 93]. Recent work has provided compelling evidence that a compromised BBB may allow entry of the human herpes simplex virus-1 (HSV-1) and with a repeated cycle of HSV-1 reactivation may lead to increased inflammation, tau hyperphosphorylation, and Aβ accumulation in the AD brain [94]. This evidence is supported by Tejeda et al. using a novel technique to detect viral DNA in whole-exome sequencing (WES) and whole-genome sequencing (WGS), where HSV-1 and human betaherpesvirus (HHV-6 A and HHV-7), identified in both blood and brain, were found to be strongly associated with AD [95]. Additionally, the COVID-19 infection, caused by the SARS-CoV-2 virus, has been associated with an increase in BBB permeability, producing a neuroinflammatory environment that resembles profiles observed in neurodegenerative Diseases such as AD, including activation of TREM1, which enhances proinflammatory signaling [96, 97]. Reduced BBB integrity has also been supported by iPSC studies, where brain endothelial cells carrying the *PSEN1* mutation for familial AD were found to exhibit alterations in adherens junctions and tight junctions, which is correlated with BBB permeability [88, 89]. The breakdown of the BBB permeability can also potentially cause migration and population of peripheral immune cells (T cells, B cells, NK cells) into the meninges and choroid plexus that make up the blood-CSF barrier of the brain parenchyma, leading to the production of cytokines and MMPs, as well as other cytotoxic factors that further disrupt the BBB and promote chronic neuroinflammation and oxidative stress, thereby increasing Aβ pathology [47, 53, 71, 98–102]. Our recent single cell transcriptome study in human brains, combined with analytic approaches to detect molecular cross-talk across cells of the BBB, identified perturbed vascular molecules and their astrocytic partners, findings that were validated through in vivo models and antemortem human imaging data [103]. Similar studies in ever expanding datasets have the potential to further unravel the molecular complexity of the BBB and its disruptions in the context of AD/ADRD.

Despite converging evidence that innate and adaptive immune responses contribute to AD pathophysiology, significant uncertainty remains regarding the temporal sequence and causal hierarchy of these processes. Distinguishing protective from maladaptive immune activation—across microglia, astrocytes, and the BBB—will require integrative, longitudinal, and cell-type–specific approaches. Continued efforts to link genetic, molecular, and imaging data are essential to clarify whether neuroinflammation is a driver, amplifier, or consequence of AD pathology.

### Oxidative stress, mitochondrial dysfunction, metal ions

#### Oxidative stress

Oxidative stress damages cell function with normal aging and contributes to the progression of multiple neurodegenerative Diseases, including AD [104, 105]. Oxidative stress occurs when there is an imbalance between free radicals, including ROS, and available antioxidants. During the production of ATP, 98% of the oxygen is consumed along the mitochondrial electron transport chain via oxidative phosphorylation, where ROS are generated continually as byproducts [106], including the production of superoxide anion radicals, a precursor to hydrogen peroxide, which is further reduced to a hydroxyl radical, the most reactive species [104, 107, 108]. While low levels of ROS are necessary for cell homeostasis and messenger functions [104], excessive levels can cause damage, as they react easily with other molecules, including neuronal proteins, lipids, and nucleic acids, potentially changing their structure and function [108, 109].

In AD, oxidative stress is evident early in the pathological cascade, with elevated markers such as 4-hydroxynonenal (4-HNE), malondialdehyde (MDA), and 8-hydroxy-2′-deoxyguanosine (8-OHdG) detected in vulnerable brain regions and in cerebrospinal fluid [109, 110]. These oxidative modifications affect key AD-related proteins, including Aβ and tau, promoting their aggregation and impairing clearance mechanisms [111]. Mitochondrial DNA damage and lipid peroxidation further exacerbate synaptic dysfunction and neuronal loss, establishing oxidative stress as both a downstream consequence and a potential driver of AD pathology [109–111].

#### Mitochondrial dysfunction

Chronic ROS attack can also lead to mitochondrial damage, including disruption of the mitochondrial respiratory chain, impairing membrane permeability and ATP production. Damage to mitochondrial DNA can cause mutations that propagate downstream mitochondrial dysfunction, increasing electron reduction of O^2^ and the formation of ROS that perpetuates a vicious cycle [104]. Much evidence for the role of mitochondrial dysfunction in AD comes from cytoplasmic hybrid (cybrid) studies. In one study, cell lines depleted of endogenous mitochondrial DNA (mtDNA) were used to create a cybrid that recapitulated specific physiologic features of AD [112]. Also, exploratory analysis using sequenced mtDNA haplogroups in two Alzheimer’s longitudinal cohorts, from the University of Kansas AD Research Center and the Alzheimer’s Disease Neuroimaging Initiative (ADNI), indicated that inherited mtDNA variants may significantly influence AD risk [113].

More recently, metabolomics studies have extended these observations by revealing coordinated alterations in brain energy metabolism in AD. Comparative analyses of AD and progressive supranuclear palsy (PSP) brains demonstrated both shared and Disease-specific disruptions in mitochondrial pathways and perturbations in amino acid and redox metabolism [114, 115]. These metabolic deficits align with impaired oxidative phosphorylation and ATP generation, consistent with transcriptomic findings of downregulated mitochondrial and ribosomal pathways in AD [115]. Collectively, studies from the Alzheimer’s Disease Metabolomics Consortium (ADMC) have reported metabolomic signatures of bioenergetic dysfunction and lipid turnover associated with AD diagnosis, progression, and genetic risk [114]. Together, these metabolomics findings highlight mitochondrial impairment as both a hallmark and potential contributor to neurodegeneration, offering promising avenues for biomarker development and therapeutic targeting.

#### Metal ions

In AD, metal ions, such as copper, zinc, and iron have been found to aggregate along with Aβ [104]. When bound to Aβ, these metal ions can catalyze the production of ROS, also leading to the damage of biomolecules [104, 107, 116]. Indeed, it has been proposed that metal homeostasis may be severely disrupted in AD brains. The concentrations of copper and zinc have been found to be three times higher in AD brains, along with increased levels of metal-Aβ aggregation, compared to healthy controls [104, 117]. Further, the degree of damage due to oxidative stress in AD brains has been found to be associated with *APOE* allele status, with *APOE4* > *APOE3* > *APOE2* [106, 118], which indicates a direct interaction between APOE4 and mitochondria that produces dysfunction along the respiratory chain [106]. Mitochondrial dysfunction may further contribute to lysosomal impairment, leading to the accumulation of autophagy byproducts, such as misfolded or aggregated proteins [119].

Collectively, dysregulated redox balance, mitochondrial injury, and metal-ion–mediated oxidative reactions interact to amplify neuronal vulnerability in AD.

### Lipid membrane dysregulation and myelin loss

#### Lipid dysregulation

Various classes of lipids, including sphingolipids, glycerophospholipids, and cholesterol [120], compose a major portion of the brain, including myelin sheath, cellular membranes, and the BBB. They serve as an energy source for impulse conduction, neurogenesis, and synaptogenesis [120] and are important for cellular signaling [121], such as the regulation of numerous ion pumps, channels, and transporters [120]. Lipids also play a role in APP processing [122] and membrane remodeling, including endocytosis, as well as membrane domains, referred to as lipid rafts, that are vital to vesicle and intracellular trafficking [121]. It is well-known that abnormal cholesterol metabolism significantly influences the development of AD.

APOE, expressed primarily by glial cells [123], has three major isoforms ε2, ε3, and ε4 that differ in lipid-binding capacity and influence on neurobiology. APOE transports cholesterol and other lipids throughout the body via APOE receptors [124]. Individuals carrying the *APOE ε4* allele have the greatest risk of developing late onset AD, a risk believed to arise from multiple interacting mechanisms, altered lipid transport and recycling, impaired clearance of Aβ and other proteins, modulation of neuroinflammation, and effects on endosomal and synaptic function. The relative contribution of each pathway remains under active investigation [124–128].

#### Myelin loss

Approximately 70 to 85% of myelin sheath is composed of lipids [129]. Oligodendrocytes, the myelinating cells of the CNS, facilitate rapid saltatory conduction of nerve impulses and help regulate the neuronal microenvironment by providing metabolic and trophic support to axons [130]. They also contribute to neuroplasticity and regenerative mechanisms, including differentiation and proliferation in response to damage [131]. Owing to their unique metabolism and physiology, oligodendrocytes are among the most vulnerable cells of the CNS, yet remain essential for neuronal survival [132]. Loss of oligodendrocytes is associated with axonal degeneration and neuronal loss, as seen in degenerative Diseases such as multiple sclerosis, and is now recognized as a major contributor to the progression of AD/ADRD [88, 130, 133, 134]. Signaling pathways such as (PI3K) and protein kinase B (AKT), for example, have been found to be dysregulated in AD/ADRD [131, 135]. Also, supporting evidence has been identified in model studies. In a study using a triple transgenic (3×Tg-AD) mouse model, Vanzulli et al. examined alterations in myelination in oligodendrocyte progenitor cells (OPCs) in an age dependent manner by immunolabeling the hippocampus of mice at 6 and 24 months of age. This model showed marked morphological atrophy at 6 months that progressed to morphological hypertrophy at 24 months [136], confirming that OPC disruption is an early pathological marker in AD. In addition to in vivo models, in vitro 3D co-culture models that include a mixture of induced neurons and primary OPCs or iPSC-derived OPCs, appropriate to AD environmental and genetic conditions, are now being developed, allowing for visualization, quantification, and the assessment of drug screening to help elucidate the role that myelination plays in AD [88, 137]. Single cell transcriptomic studies of postmortem human brain tissue have revealed that oligodendrocytes and their progenitors exhibit some of the most pronounced transcriptional and functional alterations in AD, including downregulation of genes involved in myelination, lipid metabolism, and axonal support pathways [88, 138]. These findings suggest that oligodendrocyte dysfunction is an early and pervasive component of AD pathology, potentially contributing to white-matter vulnerability, particularly in females [138]. Consistent with these cellular changes, we and others have identified downregulation of transcripts and expression networks of oligodendroglial and myelin genes in brains of patients with AD and other tauopathies [114, 139, 140].

It remains unclear whether myelin breakdown contributes to AD pathogenesis or reflects a downstream consequence of neurodegeneration [88]. Several studies support the latter interpretation, as Aβ peptides have been shown to exert cytotoxic effects on oligodendrocytes [134, 138, 141]. Neuroimaging studies have also demonstrated that white matter abnormalities are characteristic of AD, with total myelinated fiber length decreasing and myelin sheaths thinning with advancing age [142], changes that may contribute to functional white matter deficits, including impaired impulse conduction and increased vulnerability to trauma, Aβ toxicity, and oxidative stress [143]. Using magnetic resonance imaging (MRI) to compare AD and normal controls, McAleese et al. found that white matter lesions—including axon loss and demyelination, generally considered to be evidence of small vessel Disease—may also indicate cortical AD pathology [144]. Collectively, human and model studies support the notion that white matter abnormalities indicative of dysmyelination are associated with AD pathology and may serve as valuable biomarkers of Disease progression.

Together, these findings highlight the importance of lipid homeostasis and myelin integrity in maintaining neuronal health, yet the causal relationships remain incompletely understood. It is not clear whether lipid and myelin alterations act as initiating factors or downstream consequences of amyloid, tau, or vascular pathology. Addressing these questions will require longitudinal, multimodal studies that integrate lipidomics, imaging, and genetic risk data to disentangle age-related changes from AD-specific mechanisms.

### Neurotransmitter systems

Neurotransmitter imbalance, particularly acetylcholine (ACh), serotonin (5HT), norepinephrine (NE), glutamate, and Gamma-aminobutyric acid (GABA), has been implicated in AD pathogenesis.

#### Acetylcholine

The greatest emphasis for neurotransmitter involvement in AD is ACh, produced by the cholinergic diffuse modulatory system [145, 146]. ACh plays an important role in selective attention, learning, memory, perception, and consciousness [146–148]. Degeneration of cholinergic neurons [149] and reduced cholinergic transmission contribute to the memory impairments observed in AD patients [147, 150, 151]. Inhibitors of acetylcholinesterase, the enzyme responsible for ACh breakdown [145], have shown modest efficacy for symptomatic treatment [152, 153], while anticholinergic drugs that suppress ACh are associated with worsened cognitive outcomes, greater brain atrophy, and reduced cerebral glucose metabolism in older adults [151, 154, 155]. A growing body of literature supports an inverse relationship between cholinergic transmission and the pathological hallmarks of AD [146, 151]. For example, Aβ accumulation has been observed in basal forebrain cholinergic neurons in early adulthood and appears to increase in prevalence and oligomerization with aging [146, 156], potentially contributing to downstream cholinergic degeneration [146, 156].

Importantly, a genome-wide association study (GWAS) by Ramanan et al. demonstrated that genetic variation near butyrylcholinesterase, an enzyme related to ACh metabolism and present within Aβ plaques, is independently associated with cortical amyloid burden [157]. This finding highlights the relevance of cholinesterase activity not only to symptomatic treatment but also to amyloid pathology, supporting the potential of butyrylcholinesterase as both a biomarker and therapeutic target within the cholinergic-amyloid axis. Collectively, these findings reinforce the importance of ACh pathways in AD pathogenesis, particularly the basal forebrain cholinergic system.

#### Serotonin

Serotonin (5HT), another diffuse modulatory neurotransmitter and its regulators, are decreased in patients with MCI and AD [158, 159]. Positron Emission Tomography (PET) imaging studies have identified reduced serotonin transporter binding in the dorsal raphe nuclei and precuneus in individuals with MCI compared to cognitively normal controls [160]. These reductions were associated with altered functional connectivity in memory- and cognition-related regions, including the lower hippocampus, retrosplenial cortex, dorsolateral prefrontal cortex, and posterior cingulate [160, 161].

Based on these findings, selective serotonin reuptake inhibitors (SSRIs), which increase extracellular serotonin, have been explored for potential Disease-modifying effects in AD. Evidence from both human and preclinical studies has suggested that long-term SSRI use may lower amyloid burden [162]. For example, Sheline et al. showed that citalopram reduced brain interstitial Aβ levels in a transgenic AD mouse model and decreased CSF production in cognitively normal individuals after a single high dose [163]. Similarly, Bartels et al. reported a three-year delay in conversion from MCI to AD among individuals treated long-term with SSRIs for depression compared to those using non-SSRI antidepressants, though the observational nature of this study limits causal interpretation [164].

More recently, analysis of Alzheimer’s Disease Neuroimaging Initiative (ADNI) data by Terstege et al. found that SSRI use was associated with lower plasma levels of phosphorylated tau (p-tau181), a biomarker of Disease progression [165]. Notably, SSRI exposure was also linked to restored metabolic activity in the dorsal raphe nucleus (DRN), an early site of tau pathology and a critical serotonergic hub. However, cognitive outcomes were mixed across assessment methods, and prospective validation in controlled trials is still needed. Together, these findings suggest that serotonergic modulation may influence AD-related molecular processes, but clinical efficacy and durability remain uncertain [165].

Mechanistically, SSRIs and related serotonergic agents may act through various 5HT G-protein coupled receptors that regulate APP processing. Preclinical studies have reported that these receptors can activate signaling cascades such as extracellular signal-regulated kinase, enhancing α-secretase activity and reducing Aβ generation [166, 167]. In APP/PS1 mice, the 5-HT2A receptor inverse agonist, pimavanserin (Pim) reduced hippocampal Aβ [166], decrease anxiety-like behaviors, and improve memory function [166]. Receptor-specific agents such as Pim may provide more predictable outcomes than broad-acting SSRIs, though both remain at the preclinical or early clinical stage of investigation [168].

#### Norepinephrine

The noradrenergic diffuse modulatory system originates in the locus coeruleus (LC), which innervates widespread regions of the brain. Loss of NE in the cerebral cortex has been linked to increases vulnerability to AD, particularly in the early stages of the Disease [169]. Structural damage to the LC may be associated with compensatory mechanisms that elevate NE release, potentially contributing to the non-cognitive symptoms observed in AD, such as arousal, agitation and aggression [153]. Zhang et al., using mouse models, reported that Aβ can hijack NE signaling via the α2A adrenergic receptor (α2AAR), activating a pathogenic GSK3β–tau phosphorylation cascade [170]. Extending this line of research, Bueichekú et al., provided compelling longitudinal in vivo evidence in humans that reduced LC integrity precedes tau accumulation in the medial temporal lobe (MTL), and that this LC–MTL tau spreading pathway predicts future cognitive decline. Their findings support a directional model in which early LC dysfunction sets the stage for downstream tau propagation and memory impairment, underscoring the LC–NE system as a potential early therapeutic target in AD [171].

#### Glutamate

The glutamatergic system plays a critical role in synaptic transmission and plasticity and has been strongly implicated in AD pathology. Glutamate is the brain’s primary excitatory neurotransmitter, and its signaling via N-methyl-D-aspartate (NMDA) receptors is essential for learning, memory, and neuronal survival pathways [172]. However, dysregulation of the system, particularly excessive Glu release and overactivation of NMDA receptors can result in excitotoxicity, characterized by calcium influx that activates proteases, phospholipases and endonucleases, ultimately damaging cellular membranes, cytoskeletal elements, and DNA [173]. In AD, Aβ oligomers disrupt glutamate reuptake and potentiate NMDA receptor activity, amplifying calcium dysregulation, oxidative stress, and mitochondrial dysfunction [174, 175]. Astrocytic impairment in glutamate clearance further contributes to this excitotoxic environment, as demonstrated in both experimental models and human postmortem tissue [175].

To mitigate cytotoxic damage, memantine, an uncompetitive NMDA receptor antagonist approved for moderate-to-severe AD, has been evaluated as a symptomatic treatment which may suppress the extra-synaptic NMDA receptor signaling. Memantine preferentially targets excessive extra-synaptic NMDA receptor activity while sparing normal synaptic signaling, thereby reducing excitotoxic stress in preclinical models and producing modest cognitive and functional benefits in clinical trials [176]. When co-administration with the acetylcholinesterase inhibitors such as donepezil, small additive improvements in cognition and global function have been reported [177]. However, these effects are modest, do not modify underlying pathology, and long-term Disease progression remains largely unchanged. Moreover, combination therapy can increase the potential of adverse events and tolerability issues [176]. These limitations underscore the need for next-generation strategies that more precisely modulate glutamate signaling and neuronal calcium homeostasis, particularly in the prodromal or early symptomatic stages of AD.

#### Gamma-aminobutyric acid

Gamma-aminobutyric acid (GABA), the principal inhibitory neurotransmitter in the brain, has increasingly been implicated in AD pathophysiology. GABAergic signaling helps maintain neural homeostasis by regulating excitatory-inhibitory balance, synchronizing oscillatory network activity, and modulating interneuron communication across cortical and limbic structures [178]. GABAergic dysfunction may disrupt large-scale network stability and contribute to the impaired gamma oscillations and hyperexcitability observed in early AD (178). While early research emphasized glutamatergic excitotoxicity, more recent work points to a complex remodeling of GABAergic circuits that may initially serve compensatory roles but eventually contribute to cognitive decline [178, 179].

Recent work in AD mouse models has shown that somatostatin-positive GABAergic interneurons, including oriens-lacunosum moleculare (OLM) cells in the hippocampus, exhibit functional and molecular impairments. Transcriptomic profiling of these interneurons revealed downregulation of genes associated with synaptic inhibition, and targeted reactivation of their activity was sufficient to rescue memory performance in experimental settings [180]. Reductions in GABA levels and GABAergic synapse density have also been reported in both AD mouse models and human postmortem tissue, with regional variation and progressive decline observed across Disease stages [181]. Importantly, enhanced seizure activity has been observed in both humans and mouse models of AD [182, 183].

While these converging findings implicate GABAergic dysfunction as a contributor to network instability and neurodegeneration, the translational potential of targeting these circuits remains under investigation. Anti-seizure medications and other modulators of inhibitory tone are being explored in early clinical studies, but their efficacy in modifying AD progression has not yet been established. Thus, pharmacologic modulation of GABAergic signaling represents an intriguing yet still experimental avenue for therapeutic development in AD [182, 183].

### Synaptic integrity

#### Synaptic degeneration

Memory loss in AD may also be attributed to loss of synaptic integrity, which is now considered one of the strongest pathological correlates of cognitive impairment. One critical marker of synaptic health is neuronal Pentraxin 2 (NPTX2), a protein that regulates excitatory synapse function [184] by promoting the clustering and stabilization of AMPA receptors [185], particularly parvalbumin-positive interneurons [184, 186]. In AD, reduced levels of NPTX2 are strongly associated with cognitive decline, synaptic dysfunction, and Disease progression [184, 185, 187–189]. This deficiency renders synapses vulnerable to pathological processes such as Aβ-induced toxicity, tau pathology, and overactive microglia-mediated pruning [190]. NPTX2 loss disrupts the excitatory-inhibitory balance in neuronal networks, exacerbating neuroinflammation and neuronal stress, which further accelerates synaptic loss [186]. Moreover, emerging evidence suggests that NPTX2 may indirectly modulate complement-mediated synaptic pruning, providing an additional layer of protection against microglial overactivation [187, 190].

Complementing this, emerging in vivo PET imaging techniques now allow researchers to assess synaptic density through ligands such as [¹¹C]UCB-J, which binds to synaptic vesicle glycoprotein 2 A (SV2A) [191]. This has enabled direct correlations between synaptic density and cognitive performance in early AD. Mecca et al. demonstrated that global SV2A PET binding was a stronger predictor of cognitive performance than gray matter volume, with significant correlations across multiple domains, including executive function, processing speed, and language [192]. SV2A PET imaging may therefore serve as a sensitive surrogate marker for synaptic health and treatment efficacy in clinical trials.

At the molecular level, presynaptic cell-adhesion proteins known as neurexins (NRXNs) have also gained attention for their roles in synaptogenesis and their emerging links to AD pathogenesis [193, 194]. Neurexins, particularly NRXNβ isoforms, interact with Aβ oligomers at the presynaptic terminal, and this interaction appears to impair synaptic organization and promote synaptic loss [193]. Recent studies have shown that Aβ disrupts the normal binding of NRXNs to their ligands, such as neuroligins and APP, potentially blocking synaptogenic signaling and accelerating synapse degeneration [193, 195]. Genetic studies have further identified NRXN variants as contributors to AD susceptibility and progression [193].

Lastly, CSF biomarkers of synaptic integrity, especially NPTX2, SNAP25, neurogranin, and GAP43, are being actively explored for tracking Disease progression. Among them, NPTX2 is emerging as a promising target not only for its biomarker potential but also for therapeutic strategies aimed at restoring synaptic function and resilience [196].

Together, these findings underscore the vulnerability of synapses in AD and highlight the potential of integrating molecular, genetic, and imaging biomarkers to assess synaptic integrity and its therapeutic relevance.

#### Ephrin signaling pathway

The ephrin signaling pathway also plays a significant role in synaptic function and plasticity, and emerging research has begun to connect its dysregulation to AD. The ephrin receptor (Eph) family is the largest subgroup of receptor tyrosine kinases and participates in multiple processes crucial for synaptic integrity, including dendritic spine remodeling, axon guidance, and synapse formation [197]. EPHA1 (Eph receptor A1) has been associated with AD risk through GWAS; for example, Matsumoto et al. identified missense mutations in *EPHA1* linked to AD susceptibility. EPHA1 is involved in regulating neuroinflammation and BBB integrity [198], processes increasingly recognized as contributors to AD pathogenesis. Dysregulated EPHA1 signaling may enhance inflammatory responses or alter neurovascular coupling in ways that accelerate neurodegenerative changes [198].

Experimental studies suggest that dysregulation of Eph/ephrin signaling contributes to the synaptic dysfunction in AD. Using AD mouse models, Vargas et al. demonstrated that Aβ interacts with the EphA4 receptor, impairing its normal function in dendritic spine remodeling [199]. EphA4 is critical for maintaining synaptic structure, and its overactivation by Aβ has been shown to correlate with synapse loss and reduced spine density, which are strongly associated with cognitive decline in AD [199]. In pre-clinical models, pharmacologic inhibition of EphA4 signaling mitigated Aβ-associated synaptic impairment, highlighting its potential as a therapeutic target [199].

Ephrin signaling regulates the stability and density of dendritic spines, the primary sites of excitatory synaptic transmission [197]. In AD, dendritic spine loss is prominent, and disruption of Eph/ephrin pathways may contribute to this process [197, 200]. For example, overactivation of EphA receptors can promote actin cytoskeletal remodeling that destabilizes spines, whereas EphB receptors are linked to NMDA receptor function and synaptic plasticity [197]. Preliminary studies also suggest that ephrin signaling may intersect with tau pathology. Tau-associated synaptic loss is a major correlate of cognitive deficits in AD [200], and alterations in cytoskeletal dynamics influenced by ephrin pathways could potentiate tau-related synaptic damage [199]. Targeting EphA4 or other Eph receptors thus remains a promising therapeutic avenue; strategies such as small molecule inhibitors or monoclonal antibodies that modulate ephrin signaling are being investigated for their potential to stabilize synapses and mitigate Aβ- or tau-associated toxicity [201].

Collectively, these findings emphasize that synaptic dysfunction represents a central nexus linking molecular pathology to cognitive decline in AD. However, the directionality and hierarchy of these synaptic changes remain unresolved—whether they arise as early, initiating events or as downstream effects of Aβ and tau pathology. Advancing this field will require longitudinal, multimodal approaches that integrate imaging, proteomic, and functional data to clarify the temporal evolution of synaptic loss and to determine which mechanisms are most amenable to therapeutic intervention.

### Neurogenesis

Adult neurogenesis, the formation and integration of new neurons into existing neural networks [202], continues throughout adulthood in restricted neurogenic niches within the brain, including the subventricular zone (SVZ) of the lateral ventricles and the subgranular zone (SGZ) of the hippocampal dentate gyrus [202–206]. In this process, quiescent neural stem cells (NSC) become activated self-renewing, multipotent cells that generate progenitor cells capable of forming new neurons and glia [203]. New neurons of the dentate gyrus are vital for memory formation, cognition, spatial and contextual learning [204, 207, 208], as well as stress reduction via cortisol-dependent inhibition of the hypothalamic-pituitary-adrenal (HPA) axis [205], an important factor, as chronic stress may impact AD by inhibiting NSC proliferation [209].

*Intrinsic factors* include signal transduction pathways that are vital for regulation, proliferation, and survival of neuronal progenitor cells in adult neurogenesis [209], such as Wnt [210, 211], Sonic Hedgehog [212–214], Ephn [215], and Notch [216]. These mechanisms have been characterized primarily in animal and cell-based models, with emerging evidence supporting similar roles in the human hippocampus. For example, the notch signaling pathway may become disrupted by mutations in *PSEN*1, causing premature differentiation of NSC of the SVZ [211]. Another important factor, RE1 Silencing Transcription Factor (REST), influences the regulation of neurogenesis and neuronal differentiation [217, 218]. In normal aging, REST promotes stress resistance and represses genes that cause apoptosis [219, 220]. The loss of REST may contribute to dementia and the development of AD [219, 220], and a *REST* missense variant (rs3796529) has been found to be protective in AD, being associated with slower hippocampal volume loss [221]. Proteins of the Methyl-CpG-binding domain, found to occur in many Diseases including AD [222], are involved in epigenetic remodeling and influence the regulation of neurogenesis [207, 209, 223, 224].

*Extrinsic pathways* include the immune system, where long-term exposure to pro-inflammatory cytokines (i.e., IL-1β, IL-6, and TNF-α) have been shown in pre-clinical models to negatively affect SGZ neurogenesis [209]. Comparable effects have been observed in human post-mortem and in human in vitro studies reporting reduced neural progenitor proliferation in inflammatory contexts. Growth factors (i.e., BDNF, IGF, FGF-2, and PDGF) [209, 225], and vascular function also regulate and maintain the stem-cell niche, particularly as angiogenesis, the formation of new vascular networks from pre-existing vessels, is closely coupled to neurogenesis. Genes involved in angiogenesis are expressed by neural progenitor cells [204, 226], linking vascular integrity to hippocampal neurogenesis. Perturbation of these interconnected pathways, which increases with age, is believed to contribute to neurodegenerative Diseases [202, 203] including AD [209]. Hence, targeting mechanisms that preserve neurogenesis and vascular homeostasis may help sustain hippocampal plasticity and cognitive resilience in AD [227].

### Co-pathologies of AD

#### Vascular Disease

**Fig. 2:**
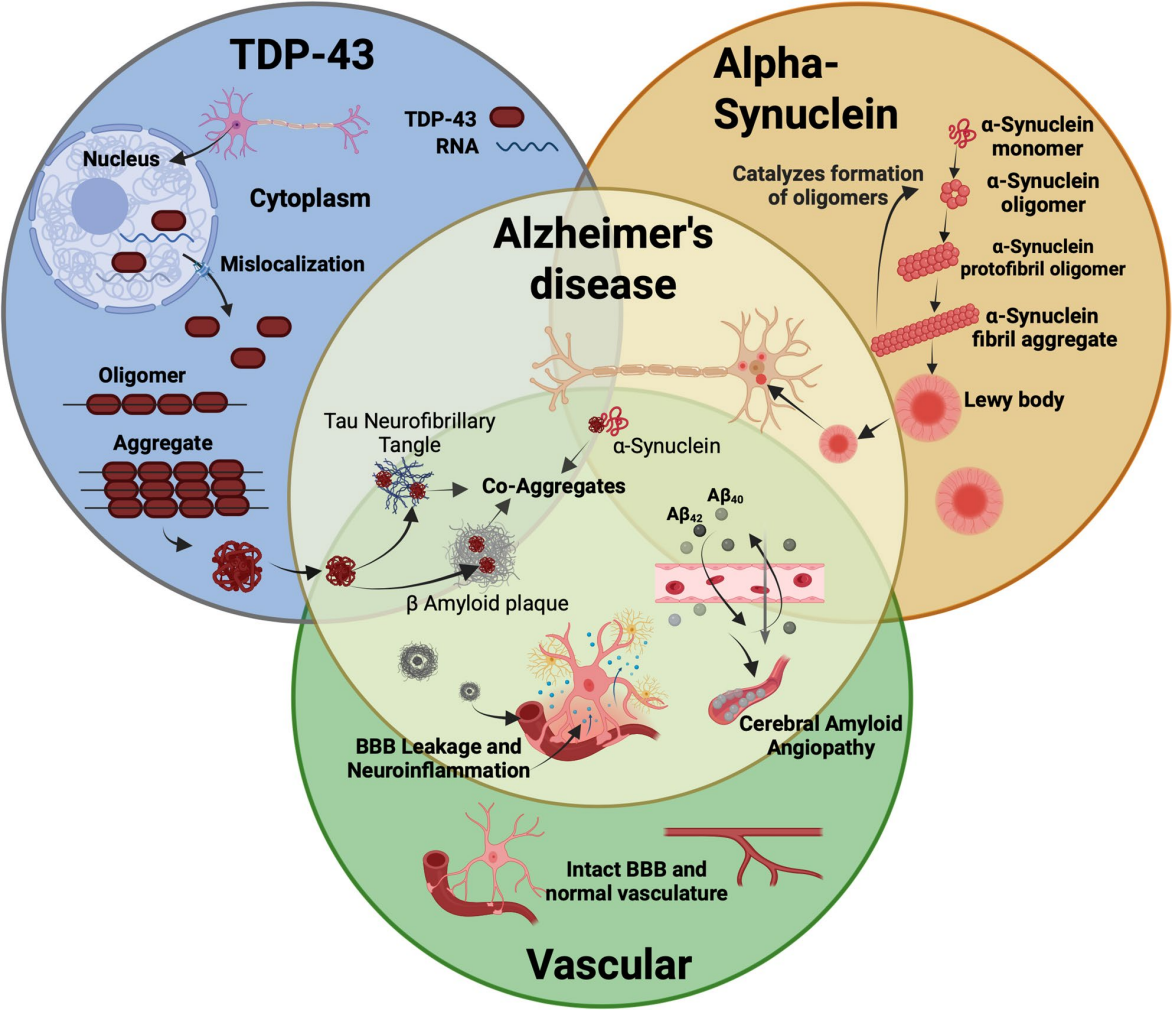
Co‑pathologies in Alzheimer’s Disease and related dementias. This schematic integrates three major co‑pathologies observed in AD/ADRD: TDP‑43 proteinopathy (blue), α‑synucleinopathy (gold), and vascular dysfunction (green). In the nucleus, TDP‑43 normally regulates RNA processing but may mis-localize to the cytoplasm, forming insoluble ubiquitinated aggregates. Separately, α‑synuclein progresses from monomers to toxic fibrils and Lewy bodies. Vascular pathology manifests as cerebral amyloid angiopathy, BBB breakdown, and neuroinflammation. Faint arrows indicate the convergence of amyloid‑β plaques, tau tangles, TDP‑43 aggregates, and α‑synuclein fibrils into central co‑aggregates. Abbreviations: TDP-43, (TAR DNA-binding protein 43); RNA, ribonucleic acid; BBB, Blood Brain Barrier; Aβ_40_, Amyloid beta 40, Aβ42, Amyloid beta 42; β Amyloid plaques, beta-amyloid plaques; α-Synuclein, alpha synuclein. Created in BioRender. Bice, P. (2025) https://BioRender.com/6vean2r.

In addition to A/T/N/I, vascular contributions to cognitive impairment and dementia (VCID) represent a major pathophysiologic pathway in late-life dementia, either as an independent diagnosis (vascular dementia, VaD) or as a contributing factor in mixed dementia (A/T/N/I(**V**) [228]. VaD typically arises from chronic or acute reductions in cerebral blood flow—due to stroke, small vessel Disease, or other perfusion abnormalities—that deprive the brain of oxygen and nutrients [228]. However, emerging evidence suggests that vascular dysfunction may not only exacerbate AD pathology but also precede and potentiate it, particularly by compromising the BBB (see Fig. 2), altering neurovascular unit integrity, and impairing perivascular clearance of Aβ and other neurotoxic metabolites [229].

Vascular dysregulation in AD has been linked to a range of pathological features, including white matter hyperintensities, microinfarcts, capillary rarefaction, and cerebral amyloid angiopathy (CAA) [228, 229]. Recent imaging and transcriptomic studies have shown that BBB breakdown in the hippocampus occurs in cognitively normal individuals at risk for AD, even before measurable Aβ or tau accumulation and correlates with early cognitive decline [230]. Additional work has demonstrated that *APOE4* carriers experience accelerated vascular injury and BBB dysfunction, providing a genetic link to early vascular vulnerability in AD [231]. Genome wide association studies of CAA by our group [232] and others [233] identified genetic loci near additional genes, highlighting novel molecular factors that may influence this vascular co-pathology in AD. Single nucleus transcriptome data from human brains, followed by functional validations in experimental models and human neuroimaging data revealed new molecular factors that are perturbed in the gliovascular unit, uncovering mechanisms of BBB dysfunction in AD [103].

Furthermore, recent Arterial Spin Labeling MRI studies have shown that regional cerebral blood flow reductions, particularly in the posterior cingulate and precuneus, precede hippocampal atrophy and correlate with early cognitive decline, supporting the view that cerebral hypoperfusion may be an early and independent contributor to AD pathogenesis [234].

Together, these findings suggest that the vascular component of AD is not merely a comorbidity but a core axis of Disease progression, influencing the onset, severity, and trajectory of cognitive decline. As such, vascular biomarkers and interventions targeting cerebrovascular health may be critical for early detection and Disease modification.

#### Alpha (α) synuclein

Substantial evidence indicates that α-synuclein, a presynaptic neuronal protein that aggregates in Lewy bodies in Parkinson’s Disease and Lewy body dementia, also co-accumulates with other proteinopathies, including Aβ and tau, in AD [235–237] (see Fig. 2). The NIA-AA research framework formally recognizes α-synuclein as part of the extended (A/T/N/I/V(**S**) classification system for staging and diagnosis [4]. Histological studies show that up to 50% of individual with AD exhibit α-synuclein pathology at autopsy, and its presence has also been detected in CSF of individuals with MCI [236]. Lewy body co-pathology is strongly associated with faster cognitive decline in AD [238], and mounting evidence supports that α synuclein, pTau, and Aβ can engage in cross seeding and synergistic fibrilization [238–240], thereby exacerbating Disease progression. Concurrent association of AD risk genetic variants with Lewy body pathology or dementia with Lewy bodies risk suggests shared pathomechanisms underlying these Diseases, which may have implications for therapautic and biomarker development [59, 60, 241, 242].

Recent work by Tosun and colleagues within the ADNI consortium has further emphasized the importance of detecting α-synuclein in vivo. Using structural MRI and machine learning approaches, their studies identified distinct neurodegenerative patterns that are predictive of Lewy body co-pathology, even in the absence of a dedicated α-synuclein PET tracer [243, 244]. These studies demonstrate that α-synuclein has independent and additive effects on brain atrophy and cognition in AD, highlighting its potential as a diagnostic and prognostic biomarker. The development of a dedicated PET ligand for α-synuclein remains a critical unmet need, not only for AD but across the broader spectrum of synucleinopathies.

#### Transactive response DNA-binding protein 43 (TDP-43)

Within the nucleus, TDP-43 is an important transcriptional regulator involved in RNA splicing, transcriptional repression, and RNA stability, as well as other essential functions in the brain [245, 246]. Proper nuclear localization of TDP-43 depends on its nuclear localization sequence (NLS) [247]. Mutations or deletions in the NLS can lead to mislocalization, where TDP-43 forms insoluble, often ubiquitinated inclusions that co-aggregate with Aβ, tau and α-synuclein in the aging brain [235, 247] (see Fig. 2). These co-aggregates are associated with worse cognitive decline in AD and ADRD [245, 248]. In AD, deposition occurs first in structures of the limbic system, including the amygdala, hippocampus, entorhinal cortex, followed by deposition in the neocortex and finally the basal forebrain [248, 249]. Data suggests that between 19 and 57% of AD have TDP-43 neural inclusions, and progression is correlated with worsening cognition and medial temporal volume loss [248]. Due to its easy accessibility in biofluids, the utility of TDP-43 as a biomarker for AD is currently being assessed. In a recent study, Zhang et al. found elevated levels of TDP-43 in the plasma of AD patients compared to cognitively normal (CN) [250]. Also, individuals who were pre-MCI (~ 2 years prior to MCI diagnosis) were characterized by high serum levels of TDP-43, whereas MCI was characterized by Aβ, and AD was characterized by Aβ and tau [251]. This finding emphasizes the potential of TDP-43 as an early blood-based biomarker for AD.

Understanding how these co-pathologies interact with AD pathology is critical for developing more effective therapeutic interventions.

### Neurometabolic dysfunction in AD

In addition to the proteinopathies that co-occur with AD, metabolic dysregulation represents a critical, yet distinct, contributor to Disease pathogenesis. Mounting evidence points to brain-specific impairments in glucose metabolism and insulin signaling, processes essential for maintaining neuronal function, synaptic integrity, and cognitive performance. These disruptions not only exacerbate classical AD pathologies such as amyloid and tau accumulation but also create a broader environment of neuronal vulnerability. The following section explores the role of insulin resistance and brain hypometabolism in AD, along with emerging therapeutic strategies aimed at restoring metabolic balance.

Insulin plays a critical role in the CNS, influencing neuronal survival, synaptic plasticity, energy metabolism, and cognition [252, 253]. Brain insulin resistance occurs when neurons fail to respond adequately to insulin, impairing synaptic function, memory formation, and neuronal survival [252]. In the insulin signaling pathway, insulin binds to its receptor (IR), activating a cascade involving insulin receptor substrates (IRS-1 and IRS-2) and downstream kinases such as PI3K and AKT [252, 254, 255]. Dysregulation of these pathways has been extensively observed in AD, leading to the accumulation of neurotoxic proteins and neuronal dysfunction [252]. For instance, increased IRS-1 phosphorylation at inhibitory serine residues (Ser616 and Ser312) disrupts downstream insulin signaling, reducing AKT activation and leading to unchecked glycogen synthase kinase-3β activity, which in turn is associated with tau hyperphosphorylation and neurofibrillary tangle formation [253, 255–258].

Glucose uptake in the brain is primarily mediated by the glucose transporter 1 (GLUT1) in astrocytes and endothelial cells and by GLUT3 in neurons. In insulin-resistant states, a decrease in GLUT1 levels has been observed in the cerebral microvasculature of AD patients, impairing glucose transport across the BBB [252]. The downregulation of neuronal GLUT3, a critical transporter for neuronal glucose uptake, disrupts energy homeostasis, contributing to synaptic dysfunction and neurodegeneration in AD [259]. Studies suggest that impaired GLUT3 function exacerbates neuronal energy deficits, contributing to hypometabolism. FDG-PET imaging studies have demonstrated reduced glucose utilization in brain regions vulnerable to neurodegeneration, further linking GLUT3 dysfunction to AD pathology [260]. This reduced glucose utilization contributes to synaptic dysfunction and neurodegeneration, further compounding the metabolic dysfunction associated with insulin resistance [260].

Insulin resistance contributes to AD pathology through multiple interconnected mechanisms, including chronic inflammation and oxidative stress. Increased microglial activation and elevated levels of pro-inflammatory cytokines (e.g., IL-6, TNF-α) exacerbate Aβ aggregation and tau pathology [256]. Additionally, excessive ROS production and mitochondrial dysfunction promote neuronal apoptosis [253]. The insulin-degrading enzyme (IDE), responsible for clearing insulin and Aβ, becomes overwhelmed in hyperinsulinemic states, contributing to Aβ accumulation and plaque formation [253, 261]. Insulin resistance also contributes to endothelial dysfunction, reduced nitric oxide production, and impaired BBB integrity, further aggravating neurovascular damage in AD [252, 256].

Given these associations, several therapeutic interventions targeting insulin signaling are being investigated. Intranasal insulin delivery is one experimental strategy that aims to bypass peripheral insulin resistance and deliver insulin directly to the brain. Early-phase clinical trials have reported modest cognitive improvements and reductions in Aβ levels in select patient subgroups, though findings remain variable, and larger, longer-term studies are needed to confirm efficacy [256, 262]. Another approach involves repurposing glucagon-like peptide-1 (GLP-1) receptor agonists, such as liraglutide, which enhance insulin signaling and reduce neuroinflammation. These compounds have demonstrated neuroprotective effects in preclinical AD models and have entered small clinical studies with preliminary but inconclusive results [256, 262–264].

Insulin sensitizing agents, including metformin and peroxisome proliferator-activated receptor agonists, are also under investigation for their potential to modulate glucose metabolism and mitigate AD-related pathology [256, 265–267]. However, data remain mixed, with some studies suggesting potential benefit while others report neutral or even adverse cognitive effects. Continued investigation into insulin signaling pathways is crucial to clarify their role in AD pathogenesis and to determine whether these metabolic interventions can yield clinically meaningful outcomes.

Collectively, these findings highlight the central role of insulin resistance and metabolic dysfunction in shaping AD pathophysiology. Yet, the direction and causality of these associations remain incompletely defined—whether disrupted insulin signaling initiates neurodegeneration or reflects downstream metabolic stress is still debated. Moreover, while early-phase trials of insulin-targeted interventions show potential, their durability, optimal timing, and population specificity remain uncertain. Integrative longitudinal studies combining metabolic imaging, genetics, and clinical phenotyping will be essential to determine whether correcting neurometabolic dysfunction can meaningfully alter the course of AD.

### Sex differences and hormones on AD pathology

Biological sex and hormonal differences also play a critical role in AD heterogeneity, contributing to Disease susceptibility, progression, and therapeutic response.

#### Sex differences

AD is approximately twice as prevalent in women as in men [268], with women accounting for nearly two-thirds of all affected individuals [225, 268]. While longer lifespan in women is often cited as a contributing factor [269], accumulating evidence points to a multifactorial basis, including sex-specific hormonal influences, genetic susceptibilities, and differences in brain aging trajectories [270].

#### Estrogen in AD

Hormones play an essential role in AD pathogenesis, particularly estrogen, which has demonstrated neuroprotective effects by reducing Aβ accumulation, modulating tau phosphorylation, and mitigating neuroinflammation [271]. Several studies indicate that declining estrogen levels, such as those occurring during menopause, hysterectomy, or oophorectomy, are associated with an increased risk of cognitive decline and neurodegeneration [272]. Notably, early menopause is linked to a more rapid cognitive decline, emphasizing the potential role of estrogen in maintaining neuronal integrity [272].

Hormone replacement therapy (HRT) has been explored as a potential intervention to mitigate cognitive decline in postmenopausal women. Some studies report that HRT is associated with improved delayed memory and increased entorhinal and amygdala volumes, particularly in *APOE4* carriers [273]. However, the efficacy of HRT remains contentious, as other studies have failed to demonstrate cognitive benefits and some have even reported accelerated decline, depending on the timing, formulation, and duration of therapy [271]. Environmental factors, such as smoking, may further influence the effectiveness of HRT, with smokers requiring higher doses of orally administered estrogen to achieve comparable clinical effects [271].

#### Progesterone and androgens in AD

Beyond estrogen, other sex hormones, including progesterone and androgens, may also contribute to AD risk. Progesterone exhibits neuroprotective effects [225, 274, 275] and has been implicated in modulating neuroinflammation, synaptic plasticity, and the growth and repair of myelin sheath [225]. The neuroactive metabolite, allopregnanolone, has been shown in preclinical studies to enhance learning and memory and reduce anxiety [225]. Androgens, such as testosterone, may exert protective effects against Aβ aggregation [276]. Declining testosterone levels in aging men or men undergoing androgen deprivation therapy have been associated with an increased risk of cognitive impairment [276]. However, the role of androgens and androgen-based therapies in AD remains largely unexplored and requires further clinical investigation.

Understanding the influence of sex hormones on AD pathology opens avenues for targeted therapeutic exploration, though this remains an area of active investigation. Selective estrogen receptor modulators (SERMs), such as raloxifene and bazedoxifene, are being evaluated for their potential to mimic estrogen’s neuroprotective effects without adverse systemic consequences [277]. Evidence to date is largely preclinical or derived from small clinical studies, and translation to clinical efficacy remains to be demonstrated. Likewise, research into sex-specific genetic risk factors, such as the differential effects of *APOE4* in men and women, highlights the need for precision medicine approaches that consider sex-specific vulnerabilities [278, 279]. Elucidating the molecular mechanisms underlying sex differences in AD pathogenesis, optimizing hormone-based therapies, and incorporating sex as a biological variable in clinical trials will be critical steps toward determining whether hormone modulation can offer clinically meaningful benefit.

These findings underscore the importance of considering sex as a biological variable in AD research and therapeutics. However, the mechanisms linking hormonal changes to Disease risk remain incompletely defined, and clinical findings on hormone-based interventions are often inconsistent due to variability in timing, dosage, and population factors. Greater integration of genomic, hormonal, and longitudinal clinical data will be critical to clarify how sex-specific pathways influence AD pathogenesis and to guide precision approaches that account for biological sex across prevention and treatment strategies.

Taken together, the preceding sections underscore the multifactorial nature of AD, where alterations in proteostasis, immune regulation, metabolism, neurotransmission, and hormonal signaling interact across biological scales. However, understanding AD as an emergent, systems-level disorder requires moving beyond individual molecular pathways toward an integrated framework that captures their collective dynamics. Systems biology provides such a framework, linking molecular mechanisms, cellular responses, and network-level interactions, to reveal how crosstalk among biological systems gives rise to Disease heterogeneity and progression.

To orient readers to the breadth, organization, and relative maturity of evidence across these biological domains, an evidence landscape summary is provided in Table 1.

**Table 1:** Evidence landscape across major systems biology domains implicated in Alzheimer’s Disease

Systems biology domain	Key pathways and mechanisms	Representative molecular features or targets discussed*	Predominant study types	Evidence maturity†
Neuroinflammation and innate immunity	Microglial activation, complement signaling, TREM2-mediated pathways, cytokine regulation	TREM2 signaling, complement cascade, microglial activation states	Human observational studies; genetics; animal and cellular models	High
Lipid metabolism and transport	APOE-mediated lipid transport, cholesterol homeostasis, membrane remodeling	APOE, cholesterol transport pathways, lipid homeostasis networks	Human genetics and biomarker studies; multi-omics	High
Synaptic integrity and neuronal signaling	Synaptic loss, vesicle trafficking, synaptic plasticity and network dysfunction	SV2A (PET imaging), NPTX2, synaptic vesicle cycling	Human imaging and CSF studies; translational models	Emerging
Proteostasis and autophagy-lysosomal pathways	Protein misfolding, degradation, lysosomal dysfunction, autophagy	HSP70, HSP90, autophagy–lysosomal pathways, protein clearance mechanisms	Preclinical models with growing human biomarker evidence	Moderate
Mitochondrial dysfunction and oxidative stress	Bioenergetic failure, ROS generation, redox imbalance	Mitochondrial bioenergetics (oxidative phosphorylation), oxidative damage pathways	Preclinical studies; limited human biomarker data	Moderate
Vascular and blood–brain barrier dysfunction	Cerebrovascular integrity, BBB transport, neurovascular coupling	Neurovascular coupling, cerebrovascular and BBB transport mechanisms	Human imaging and neuropathology studies	Emerging
Genomics, transcriptomics, and multi-omics integration	Polygenic risk architecture, cell-type–specific gene regulation, network-based dysregulation	GWAS loci, cell-type–specific molecular networks	Large-scale human cohort studies; integrative multi-omics	High

†Evidence maturity reflects the relative depth, consistency, and translational relevance of the current literature as synthesized in this scoping review

The table provides a high-level synthesis of biological pathways, representative molecular features, predominant study types, and relative evidence maturity across domains discussed in this scoping review. The intent is to orient readers to the current evidence base rather than to provide an exhaustive catalog of individual studies.

### Systems biology approaches in AD

#### Integrative pathway and network-level analysis

Understanding the complex network of biological pathways in AD requires more than cataloging isolated molecular changes; it demands a unifying framework that captures how genetic, epigenetic, transcriptomic, proteomic, metabolomic, microbiomics, and environmental signals converge to shape Disease trajectories [21, 85]. Within this context, systems biology provides both a conceptual and computational bridge by leveraging multi-omics data to construct and test models of interacting molecular networks. By mapping the multi-layered interactome, which spans genomic regulation, molecular signaling, and cellular responses, these data-rich strategies enable researchers to examine how perturbations in one domain influence others and to identify convergent nodes potentially amenable to intervention [280]. Importantly, omics technologies can reveal subtype-specific patterns, temporal staging of pathology, and unexpected crosstalk among biological systems (e.g., lipid metabolism and immune signaling) [281]. A systems-level perspective grounded in pathway and network analysis thus supports precision strategies by connecting biological mechanisms to measurable clinical phenotypes and generating hypotheses for future validation.

#### Functional genomics

**Fig. 3:**
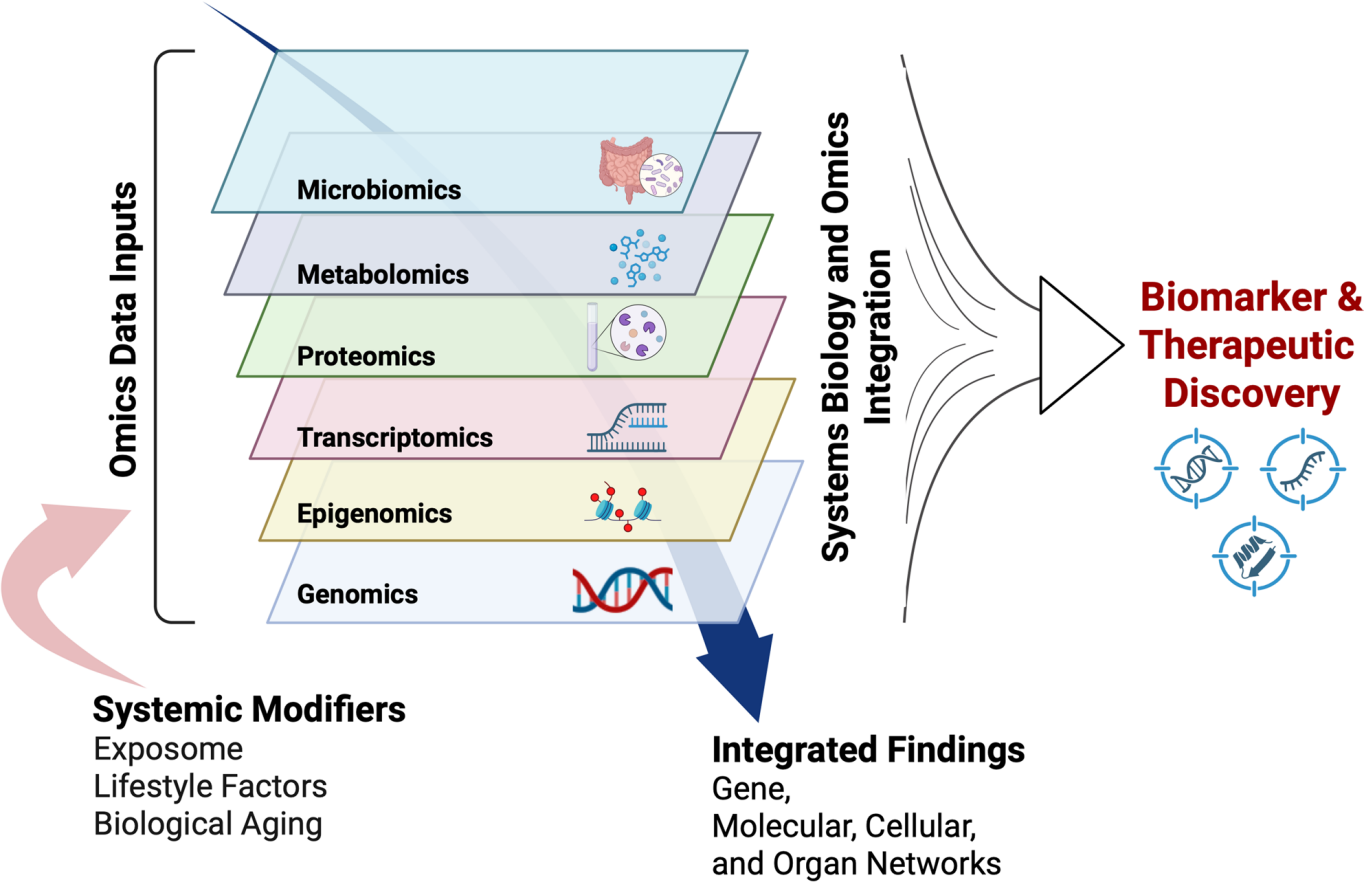
Multi-omics integration of systems biology approaches in Alzheimer’s Disease biomarker and therapeutic discovery. This conceptual framework illustrates how multi-omics data, spanning genomics, epigenomics, transcriptomics, proteomics, metabolomics, and microbiomics, are integrated and analyzed to uncover biologically meaningful patterns associated with AD. Each omics layer contributes to the mapping of gene, molecular, cellular, and organ-level networks, which collectively define the AD phenotype. The integration and analysis of these data layers support the identification of molecular subtypes and Disease mechanisms. Systemic modifiers such the exposome, lifestyle factors, and biological aging influence molecular expression across omics layers and contribute to heterogeneity in Disease risk and progression. Created in BioRender. Bice, P. (2025) https://BioRender.com/fo5sno1.

To understand how these pathways operate across biological scales, functional genomics approaches investigate genome-wide interactions and expression patterns that contribute to phenotypic heterogeneity in AD [17, 18]. These methods have become increasingly valuable in identifying novel pathways implicated in Disease pathology by integrating expression profiling, structural analyses of genes and proteins, and intragenomic interactions on a global scale [282]. Functional genomics determines how the components of a biological system work together dynamically—across Disease stages—to produce specific phenotypes, thereby uncovering critical targets for further research and therapeutic intervention [283]. Multiple layers of functional omics can be explored, including genomics, epigenomics, transcriptomics, proteomics, metabolomics, and microbiomics (see Fig. 3).

### Multi-omics platforms in AD

Systems biology draws strength from layered, high-throughput omics technologies. The following sections detail contributions of each omics platform to our understanding of AD.

#### Genomics

The field of modern genetics expands classical/Mendelian genetics, which focuses on a small number of genes and inherited traits, to the study of whole genomes and a full complement of hereditary material, utilizing research data from GWAS, WGS, and WES, and more recently, large scale genome-wide association by proxy (GWAX). Advances in bioinformatic analytical methods for genomic data have led to the identification of more than 600 candidate genes for AD [8], as well as 76 loci with genome-wide significant evidence for AD risk validated by the Alzheimer’s Disease Sequencing Project (ADSP) [284]. AD risk and protective genes include *APP*,* PSEN1*, and *PSEN2,* which harbor well-established dominantly inherited mutations that serve as biomarkers for early onset familial AD (EOFAD) [16] as well as rare protective variants [285]. Coding mutations in *PSEN1* and *PSEN2* interfere with the function of the γ-secretase complex, altering the processing of APP and leading to toxic forms and/or the overproduction of Aβ [286, 287], causing nearly 100% penetrance and inevitable cognitive decline [286]. The *APOE* gene *ε4* allele, which is associated with reduced clearance of Aβ and many other proposed pathway disturbances, is the most common genetic risk factor for AD [104], where a single *ε4* allele increases the risk of AD by 2 to 3-fold, and two alleles increase the risk by 10 to 15-fold [288] in non-Hispanic white populations [289], with variable effects in non-white ethnoracial groups. Conversely, *APOE ε2*, identified as a longevity gene, is associated with a 50% decreased risk relative to *APOE ε3* [288, 290], which is normally considered as a neutral allele in AD. Interestingly, a case study by Arboleda-Velasquez et al. suggests that a rare form of the *APOE ε3* allele, the *APOE3 Christchurch* (*APOE3ch* or R136S) mutation, confers protection (significant delay of onset) against the autosomal dominant Alzheimer’s Disease *PSEN1* gene mutation [291]. This study found that, homozygosity of the *APOE3ch* allele was associated with high Aβ deposition but limited tau neurofibrillary tangles and neurodegeneration [288, 291]. In studies using mouse models for AD, Liu et al. identified an *APOE ε3* Jacksonville variant (*apoE3*-Jac or V236E) that reduces apoE self-aggregation, including that of apoE4, thereby improving its lipidation capacity [288, 292–294], which was associated with decreased Aβ deposition and increased cholesterol efflux [292]. The Colombia–Boston biomarker research study (COLBOS) identified another rare variant, *RELN-COLBOS*, in a male participant heterozygous for the *PSEN1-E280A* mutation [295]. The *RELN* variant, which codes for the reelin protein, was associated with delayed age and onset of cognitive decline (MCI diagnosis at age 67) [295]. Reelin has been shown to be involved in numerous brain processes, including nerve cell migration [295]. Another rare variant, R251G, observed predominantly in individuals carrying the *APOE ε4* allele, has also been associated with reduced risk of AD. R251G is located in the lipid-binding region of APOE and may impair its ability to form insoluble oligomers [294]. Together, these findings may prove pivotal in understanding the pathophysiology of AD and impact therapeutic development [293].

Early large-scale GWAS, including the landmark meta-analysis by Lambert et al. [296], provided the first robust evidence that common variation contributes to LOAD. These early studies identified a small set of loci, including *CLU*, *CR1*, *ABCA7*, *BIN1*, *CD33*, *CD2AP*, *EPHA1*, *MS4A6A–MS4A4E*, and *PICALM* [296]. These discoveries were pivotal in demonstrating that immune regulation, lipid metabolism, and endocytosis are key biological pathways underlying AD susceptibility.

Building on this foundation, subsequent GWAS, GWAX, and large-scale meta-analyses have dramatically expanded the genetic architecture of AD, now identifying dozens of loci and hundreds of candidate genes. A major advance during this period was the identification of TREM2 through targeted and sequencing-based approaches, marking a shift from discovery driven primarily by common variants to one incorporating rare functional alleles. The *TREM2* R47H missense variant increases AD risk [57, 71, 297–300] by approximately 2-3-fold [73], whereas putative regulatory variants that enhance *TREM2* expression appear protective [58, 297], highlighting the central role of microglial activation, phagocytosis, and innate immune signaling in AD pathogenesis.

Further expansion of AD risk loci came from proxy-based approaches, such as the work by Marioni et al. [301], which leveraged parental family history of AD to increase statistical power. This method identified additional loci, including *ACE*, *ADAM10*, *BCKDK/KAT8*, *VKORC1*, and *KANSL1*. Large-scale meta-analyses by Jansen et al. [302] and Kunkle et al. [303] later confirmed and extended these findings, implicating more than 25 genome-wide significant loci and highlighting genes such as *ADAM10*, *ADAMTS1*, *IQCK*, and *WWOX*. These studies reinforced the importance of pathways involving APP processing, lipid metabolism, endocytosis, and immune function.

Most recently, Nho et al. conducted the first large GWAS meta-analysis of tau PET as an AD endophenotype, identifying rs2113389 at the *CYP1B1–RMDN2* locus, which explained 4.3% of cortical tau variance, exceeding the contribution of *APOE ε4* [304]. This finding underscores the value of imaging-genetics approaches for refining mechanistic understanding and mapping genetic influences onto specific molecular processes and pathologies.

Together, these advances illustrate how AD genetics has progressed from an initially few genes to a broad, systems-level architecture encompassing common and rare variation across diverse biological pathways. While genetic studies have yielded major insights into AD susceptibility, they also highlight that genetic risk alone does not fully account for Disease heterogeneity, motivating expanded efforts in epigenomics and transcriptomics to capture the regulatory mechanisms that modulate gene expression across the Disease continuum.

#### Epigenomics

Ongoing research is continually discovering how epigenetic factors impact complex Diseases, including AD. Epigenetics allows for modifications in gene expression that are not due to modifications in DNA sequence, allowing for a change in phenotype without a change in genotype [305]. Epigenetic modifications are initiated and sustained by DNA methylation, histone modification and non-coding RNA gene silencing [306]. Over the past decade, there has been increasing evidence of epigenetic mechanisms in synaptic plasticity, learning, and memory [307, 308], and epigenetic mechanisms may play a crucial role in DNA repair [309–311]. More recently, an epigenome-wide association study (EWAS) was performed on a subset of ADNI participants (*n* = 653) diagnosed with either AD or MCI vs. healthy controls to assess novel blood-based biomarkers in AD. A full range of omics data was available on all participants. The study examined differential methylated positions in AD vs. CN or MCI and MCI vs. CN. The strongest differential methylated positions were annotated to specific genes within each subgroup, including *FAM8A1* (AD vs. CN), which is important in ubiquitination of proteins associated with AD; *CLIP4* (MCI vs. CN) is a paralogous gene that may be involved in microtubule binding, and *NUCB2* (AD vs. MCI), which encodes a calcium binding protein that regulates intracellular calcium [312]. These studies support the role of epigenomics in identifying blood-based biomarkers and critical pathways in the progression of AD.

#### Transcriptomics

Transcriptome-wide association studies (TWAS) are an extension of GWAS and serve to characterize the transcriptional activity of the entire genome or a subset of target genes, including all coding and non-coding regions. Essentially, the transcriptome represents a snapshot of gene expression under specific circumstances at a given time. When integrated with genomic data, transcriptome analysis can help nominate the Disease gene at a GWAS locus, implicate transcriptional dysregulation as the mechanism of action of the Disease risk variant(s), and suggest whether the effect of a candidate transcript is likely to be beneficial or detrimental [313–316]. It is also apparent that the complex mechanisms associated with regulation/dysregulation of gene expression, including the interactions between transcription factors, alternative splicing patterns, and coding and non-coding RNAs play a significant role in the pathogenesis of AD [317–320]. Of particular interest is CREB5, a protein involved in the regulation of gene expression and neuroplasticity. In response to cell signaling, CREB5 binds to the cAMP response element (CRE) to induce CRE-mediated gene transcription [321]. cAMP is involved in a variety of cellular processes, including cell growth and differentiation, as well as gene transcription and protein expression [322]. Another interesting candidate is *TMBIM6*, a gene that codes for Bax inhibitor 1, a protein that protects the cell from stress induced apoptosis, modulates unfolded protein response signaling [323] helps to maintain Ca^2+^ homeostasis of the endoplasmic reticulum [323] and has been shown to support neurogenesis [324]. Using RNA from peripheral blood, Nho et al. examined gene expression in LOAD and found that abnormal gene expression may promote the onset and progression of AD [325]. Nho et al. also identified five genes that were differentially expressed, including four genes that were upregulated, *CREB5*,* FLOT1*,* CD46*,* IRAK3*, and *TMBIM6* and one that was downregulated, *RPAIN*, which was not previously identified in AD research [325]. *RPAIN* codes for a protein involved in the transport of replication protein A, a binding protein important for DNA replication, repair, and recombination [325, 326]; downregulation may reduce DNA repair efficiency and lead to cell cycle dysregulation [325]. Another study [327] highlights the importance of transcriptomics in precision medicine for AD. Using the Mount Sinai/JJ Peters VA Medical Center Brain Bank (MSBB-AD), the authors identified clinical and transcriptomic signatures from individuals diagnosed with AD and replicated these findings in the Religious Orders Study–Memory and Aging Project (ROSMAP). Differential gene expression across four brain regions revealed the para-hippocampal gyrus as the most vulnerable region. Multiscale network analysis further identified three major molecular subtypes of AD, each defined by distinct transcriptional programs and genetic regulators. Notably, some variation among AD mouse models mirrored the human subtypes, suggesting that animal models may preferentially reflect specific Disease subtypes. As the authors emphasize, this may help explain why therapeutics developed in preclinical models often fail in clinical trials, as a treatment effective in a model representing one AD subtype may not translate to efficacy across all subtypes in heterogeneous human populations [327]. These findings underscore the importance of a precision medicine approach, where therapeutic strategies are tailored to molecularly defined subgroups of AD patients to improve treatment outcomes.

#### Proteomics

Proteomics provides critical information about the functional proteins within a cell or organism, including post-translational modifications and protein-protein interactions, allowing researchers to investigate the dynamic aspects of cellular processes, including how genes are ultimately translated under varying conditions and across multiple Disease stages [328–330]. For example, Bai et al. used deep layer proteomic analysis to identify and validate altered key proteins and phosphoproteins in stage-associated molecular networks in AD, where comparisons of CSF and brain tissue proteomes revealed several biomarker candidates [331]. In another study, multiple proteins involved in the regulation of biological processes downstream of Aβ and tau deposition, including the inflammatory response, apoptosis, and endocytosis, were identified and validated in both CSF and plasma, where approximately one third of the proteins found in CSF were significantly correlated with their plasma analogs, and several differentially regulated proteins were associated with cortical atrophy and cognitive performance [332]. Notably, plasma biomarkers were able to successfully distinguish AD dementia and prodromal AD from CN [332]. In a mechanistic study, Xiong et al. examined differences in the proteomic profiles of amyloid plaques in AD and age matched non-AD brains. Though 40 proteins were enriched in both AD and non-AD amyloid plaques, including APOE, specific synaptic structural proteins, including C1r, C5, and C9, were upregulated in AD cases only, emphasizing the involvement of complex and adaptive proteostasis networks in AD progression [333]. This finding is particularly important because it may explain the poor correlation between plaque burden and cognitive impairment that undermines the amyloid beta hypothesis in AD. It may also provide a plausible explanation for why therapeutics that target amyloid plaques have been less than satisfactory [333]. Thus, proteomic applications not only have the potential to identify critical biomarkers and pathways in AD but also deepen our mechanistic understanding of the Disease.

#### Metabolomics

Metabolomics is an emerging and powerful tool involving the comprehensive study of small molecules, including hundreds to thousands of metabolites, found within cells, biofluids, and tissues [334]. The interaction of these small molecules within a biological system are collectively known as the metabolome [335]. Many biochemical processes are affected during the progression of AD and impact brain metabolism. Studying metabolic profiles may offer a more rapid method for biomarker discovery and validation. For example, using integrative network analysis in a large-scale metabolomics study, Horgusluoglu et al. identified novel metabolites highly associated with clinical outcomes in AD, including short-chain acylcarnitines/amino acids and medium/long-chain acylcarnitines, as well as their upstream regulators, *ABCA1* and *CPT1A*, both of which code for proteins important for the neuronal system and the immune response [336]. Mutations in *ABCA1* can reduce APOE lipidation, increasing amyloid plaques and reducing cholesterol efflux, thereby causing Aβ overproduction, transport abnormalities, and gene expression changes [293]. In another study, Toledo et al. [334] used metabolomics to identify peripheral metabolic changes in blood serum samples from ADNI participants. The metabolic data, along with neuroimaging and cognitive performance measures, showed that changes in lipid metabolism are associated with early stages of Disease, while changes in mitochondrial energetics and energy utilization are associated with later stages of Disease, thereby linking metabolism to the biochemical trajectories of Disease using the established temporal sequence of pathophysiological stages of AD. Together these types of studies illustrate the feasibility of using an integrative systems biology framework [336] and provide strong evidence that metabolomics is a powerful and efficacious method for identifying targets for AD pathogenesis [334].

#### Microbiomics

Microbiomics is a fast-growing field that investigates specific microbial communities–the microbiota–and how they change over time or under particular physiologic conditions or environments (e.g., human gut, mouth, whole organism). The gut microbiota consists of the total bacteria, viruses, fungi, and microscopic protozoa that inhabit the gastrointestinal tract [337, 338].

Increasing evidence indicates bidirectional communication between the intestinal microbiota and the brain [337, 339], collectively referred to as the gut-brain-axis, which includes the CNS, the enteric nervous system, and the gastrointestinal tract [340]. This crosstalk is hypothesized to influence neurological health and Disease. Over the past decade, numerous studies have reported associations between alterations in the gut microbiome and AD [341], Parkinson’s Disease [342], multiple sclerosis [343], and stroke [344].

The intestinal microbiotas interact with the CNS through neural, endocrine, and immune pathways [337, 345]. For example, microbiota can produce neurochemicals analogous to neuroendocrine hormones or neurotransmitters [346], including ACh, NE, serotonin, GABA, and dopamine [337, 347] –and microbial metabolites or toxins may influence host physiology, particularly in vulnerable populations (e.g., very old or very young) [337]. Experimental studies in mice suggest that specific bacterial strains, such as *Escherichia coli*, can modulate the HPA axis [337, 340], which regulates stress responses and inflammatory tone [345], though the translatability of these findings to humans remains uncertain.

Observational studies have also linked oral microbiota (e.g., chronic periodontitis) with systemic inflammation and AD risk [348–350]. Overall, current evidence supports a correlative relationship between microbiome alterations and neurodegenerative Disease phenotypes, but causal mechanisms and directional effects remain to be established. Nevertheless, microbiome signatures may offer promising biomarker candidates for Disease stratification and for evaluating therapeutic interventions targeting the gut-brain-axis.

### The exposome and lifestyle factors

While the biological mechanisms underlying AD are central to its pathology, the role of lifestyle and exposome factors provides critical context for understanding the broader systemic influences on Disease progression. External environmental exposures and internal modifiable risk factors interact with the molecular pathways discussed above, adding another layer of complexity to the systems biology framework for AD.

The exposome was first proposed by Christopher Paul Wild in 2005 [351] as a necessary complement to multi-omics studies. The exposome represents the total of lifetime exposures from conception onward and how those exposures impact health [351–353]. Because individual differences play a significant role in cognitive loss, assessing environmental factors is essential for understanding the pathophysiology of AD [352]. In fact, twin studies have shown that as much as 30% of the risk of developing AD may be environmental, emphasizing the importance of non-genetic risk factors in AD [354, 355]. External factors, such as air pollution [356, 357], socioeconomic status/education and neighborhood social stressors [358–361] have been shown to increase the risk for developing AD, as well as internal factors, that include diet and lifestyle [362], sleep/circadian rhythm disruption [8, 320, 363–366], pathological conditions, like obesity [367], type 2 diabetes [368], traumatic brain injury [369], and the aforementioned microbiota [337]. In a large proof-of-concept clinical trial, the Finnish Geriatric Intervention Study to Prevent Cognitive Impairment and Disability (FINGER), Ngandu et al. found that vascular and lifestyle intervention was able to improve or maintain cognitive function in at-risk elderly [370], and the model is currently being adapted and optimized to promote implementable prevention strategies in the U.S., referred to as the U.S. Study to Protect Brain Health Through Lifestyle Intervention to Reduce Risk (U.S. POINTER) [371]. In its 2017 report, the National Academies of Sciences, Engineering, and Medicine (NASEM) reviewed lifestyle interventions for preventing cognitive decline and found the evidence for certain factors, such as physical activity, blood pressure management, and cognitive training, encouraging but inconclusive at the time [372]. However, more recent NIA-funded studies have strengthened this view, showing that a combination of five healthy lifestyle behaviors, including physical activity, smoking avoidance, moderate alcohol use, a high-quality diet, and cognitive engagement, was associated with a substantially reduced risk of AD [373]. Together this evidence highlights the importance of investigating the role of modifiable lifestyle risk factors in AD, particularly for Disease prevention or delay of onset.

Across these interconnected molecular and environmental domains—from proteostasis and immune regulation to lipid metabolism, synaptic integrity, neurometabolic function, hormonal modulation, and lifestyle exposures—a consistent theme emerges: AD reflects a convergence of multifactorial perturbations rather than isolated defects. Integrating these molecular and exposome insights through systems biology offers a powerful means to map how diverse cellular processes and environmental influences interact to drive network-level dysfunction and clinical decline. The next frontier lies in translating this integrative framework into predictive, testable models that connect molecular mechanisms and environmental modifiers to Disease trajectories and therapeutic responsiveness.

### Future directions

Advanced tools are critical for uncovering the underlying pathogenic mechanisms of AD. High-throughput single-cell transcriptomics, which enables researchers to analyze gene expression patterns in individual brain cells, offers insight into how specific cell types—such as neurons, microglia, and astrocytes—are differentially affected across AD stages. This includes capturing activation states of immune cells, such as microglia, that influence neuroinflammation [374]. Spatial omics technologies, including in situ hybridization and spatial transcriptomics, further extend these capabilities by providing spatially resolved gene expression data within brain tissue, elucidating cell-cell interactions and the distribution of Disease pathology [375]. The integration of longitudinal studies using data from diverse cohorts will provide a more comprehensive view of Disease progression, leading to better prediction capabilities and increasing replication and the generalizability of results [341, 376–378].

Artificial intelligence (AI) is poised to revolutionize AD research by advancing biomarker discovery and predictive modeling. Machine learning algorithms can process vast multi-omics datasets to uncover novel biomarkers linked to Disease onset and progression, providing new therapeutic targets. AI-powered predictive models can integrate clinical, genetic, and imaging data to forecast individual trajectories of cognitive decline, enabling earlier and more precise diagnoses [379, 380]. When combined with systems biology frameworks, these computational approaches enable the formulation and testing of network-level hypotheses—for example, predicting how perturbations in lipid metabolism or neuroinflammatory signaling alter cellular networks and cognitive outcomes. These innovations hold promise for personalized treatment strategies, improved clinical trial design, and the advancement of precision medicine in AD/ADRD.

### Limitations

This review has several limitations inherent to its scoping design. First, literature searches were conducted primarily through PubMed, supplemented by targeted Google and Google Scholar queries, which may have excluded relevant studies indexed in other databases. Second, although ASReview’s active-learning framework was used to validate coverage and prioritize relevance, scoping reviews involve iterative refinement, and additional studies may emerge as the literature evolves. Third, given the heterogeneity of methodologies and the variable maturity of evidence across biological domains, quantitative synthesis was not feasible, and findings are limited to qualitative integration. Finally, screening and study selection were conducted by a single reviewer, which may introduce subjective bias despite the use of ASReview to enhance prioritization; moreover, because evidence maturity varies widely across domains, some emerging areas could only be summarized at a high level, whereas hallmark AD pathologies benefit from a more established evidence base.

## Conclusion

Alzheimer’s Disease is a multifactorial neurodegenerative disorder in which diverse biological pathways converge to drive pathology and clinical decline. As summarized in this review, these mechanisms differ in their empirical maturity, with hallmark pathologies grounded in long-standing evidence and newer systems-level fields offering promising but still developing insights. While Aβ deposition and tau hyperphosphorylation remain central to Disease characterization, numerous complementary mechanisms, including neuroinflammation, metabolic dysregulation, and synaptic dysfunction, contribute to Disease heterogeneity. Systems biology offers a unifying analytical framework for integrating these mechanisms across molecular, cellular, and network levels. By modeling interactions among genetic, epigenetic, proteomic, metabolomic, and environmental factors, systems-level approaches support the development of mechanistically grounded, testable hypotheses about Disease initiation, progression, and therapeutic intervention points. This integrative strategy moves the field toward predictive models of AD pathogenesis and biomarker-driven precision therapeutics tailored to individual molecular profiles.

Of critical importance for prevention and precision medicine is understanding the mechanisms and pathways that underlie plaque and tangle formation, maintenance and clearance, which are likely to differ across individuals. By incorporating these processes into computational and network-based models, systems biology enables the identification of causal pathways, feedback loops, and nodes of therapeutic opportunity. Emerging tools, concepts and knowledge pave the way for next-generation biomarker discovery and precision medicine strategies tailored to the unique molecular profiles of individual patients.

## Supplementary Information

Below is the link to the electronic supplementary material.

Supplementary Material 1

## Data Availability

No datasets were generated or analysed during the current study.

## Abbreviations

5HT: Serotonin
ACh: Acetylcholine
AD: Alzheimer’s Disease
ADNI: Alzheimer’s Disease Neuroimaging Initiative
ADRD: Alzheimer Disease and related dementias
AI: Artificial Intelligence
AKT: Protein kinase B
APOE: Apolipoprotein E
APP: Amyloid precursor protein
A/T/N: Amyloid, Tau, and Neurodegeneration
Aβ: β-amyloid
BBB: Blood brain barrier
CN: Cognitively normal
CRE: cAMP response element
Eph: Ephrin receptor family
FINGER: Finnish Geriatric Intervention Study to Prevent Cognitive Impairment and Disability
GABA: Gamma-aminobutyric acid
GLUT1: Glucose transporter 1
GWAS: Genome-wide association studies
GWAX: Genome-wide association by proxy
HHV: Human betaherpesvirus
HPA: Hypothalamic-pituitary-adrenal
HRT: Hormone replacement therapy
HSPs: Heat shock proteins
HSV-1: Human herpes simplex virus-1
I: Inflammation
IFN: Type 1 interferon
IRS: Insulin receptor substrates
LOAD: Late onset Alzheimer’s Disease
MMPs: Metalloproteinases
MRI: Magnetic resonance imaging
MSBB-AD: Mount Sinai Brain Bank-AD
mtDNA: Mitochondrial DNA
NE: Norepinephrine
NPTX2: Pentraxin 2
NSC: Neural stem cells
PI3K: Phosphoinositide 3-Kinase
Pim: Pimavanserin
PLCG2: Phospholipase C-γ2
pTau: Hyperphosphorylated tau
RAGE: Receptor for advanced glycation end products
REST: RE1 Silencing Transcription Factor
ROS: Reactive oxygen species
ROSMAP: Religious Orders Study–Memory and Aging Project
SERMs: Selective estrogen receptor modulators
SGZ: Subgranular zone
SSRIs: Selective serotonin reuptake inhibitors
SV2A: Synaptic vesicle glycoprotein 2 A
SVZ: Subventricular zone
TDP-43: Transactive response DNA-binding protein 43
TNFα: Tumor necrosis factor
TWAS: Transcriptome-wide association studies
U.S. POINTER: U.S. Study to protect brain health through lifestyle intervention to reduce Risk
UPS: Ubiquitin-proteasome system
V: Vascular
VaD: Vascular dementia
WES: Whole-exome sequencing
WGS: Whole genome sequencing
