# Supplementary material for: Systems biology of Alzheimer’s Disease: a scoping review of key pathways and mechanisms
Source: Mol Neurodegener. 2026 Feb 23;21:21. doi: 10.1186/s13024-026-00934-4 (PMC13032323; doi:10.1186/s13024-026-00934-4)
Supplement: Supplementary file 1 — Supplementary Material 1 [file 13024_2026_934_MOESM1_ESM.docx]

**Supplemental Material**

**Supplementary Table S1. Descriptive characteristics of studies included in this scoping review.**

| **Study characteristic** | **Summary description** |
| --- | --- |
| **Scope of included studies** | Studies spanning multiple systems biology domains relevant to Alzheimer’s disease |
| **Primary study type** | Predominantly human observational and cohort-based studies, with complementary preclinical and translational investigations |
| **Sources of evidence** | Peer-reviewed literature identified primarily through PubMed, with targeted supplementation from authoritative online resources |
| **Biological domains represented** | Neuroinflammation, lipid metabolism, synaptic integrity, proteostasis and autophagy-lysosomal pathways, mitochondrial and oxidative pathways, vascular and neurovascular mechanisms, and genomics/multi-omics integration |
| **Primary analytical modalities** | Genomics and transcriptomics, proteomics, metabolomics, neuroimaging, and integrative multi-omics approaches |
| **Study design** | Primarily cross-sectional studies, with a subset of longitudinal investigations |
| **Level of biological organization** | Molecular, cellular, systems, and network-level analyses |
| **Translational focus** | Biomarker discovery, mechanistic insight, risk stratification, and disease heterogeneity |
| **Overall evidence profile** | Heterogeneous evidence base with variable maturity across domains, consistent with a scoping review framework |

This table provides a descriptive summary of study characteristics across included literature and is not intended as an exhaustive or quantitative inventory of individual studies.

**Supplementary Table S2. Domain-specific machine-learning–assisted screening results using ASReview.**

| **Domain / Dataset** | **Initial Dataset Size** | **Records Screened Before Plateau** | **Relevant (Included)** | **Not Relevant** | **Unlabeled (Remaining)** | **Matches with Manuscript References** |
| --- | --- | --- | --- | --- | --- | --- |
| **Copathology** (TDP-43, α-synuclein, vascular, mixed dementia) | 4713 | 1800 | **451** | 2204 | 2058 | **15** |
| **Multi-Omics** (genomics, transcriptomics, proteomics, metabolomics, microbiome) | 5325 | 1265 | **428** | 837 | 4060 | **33** |
| **Immune / Oxidative Stress** | 7048 | 1842 | **549** | 1293 | 5206 | **33** |
| **Hallmark Pathology** (Aβ, tau, plaques, tangles, staging, seeding) | 5986 | 1712 | **653** | 1059 | 4274 | **20** |
| **Synaptic Integrity / Neurotransmission** | 3618 | 803 | **323** | 480 | 2815 | **17** |
| **Total Across All Domains** | **26690^*^** | — | **2404** | — | — | **118 unique non-overlapping references across domains** |

^*^ Because each domain was retrieved through an independent PubMed search, some records may appear in multiple domains.

The table summarizes initial dataset sizes, screening depth before plateau, and the number of studies identified as relevant across five predefined biological domains. These results provide a machine-learning–based validation of the manual screening process and support reproducibility of the search strategy.
